# High SARS-CoV-2 seroprevalence in children and adults in the Austrian ski resort of Ischgl

**DOI:** 10.1038/s43856-021-00007-1

**Published:** 2021-06-30

**Authors:** Ludwig Knabl, Tanmay Mitra, Janine Kimpel, Annika Rössler, André Volland, Andreas Walser, Hanno Ulmer, Lisa Pipperger, Sebastian C. Binder, Lydia Riepler, Katie Bates, Arnab Bandyopadhyay, Marta Schips, Mrinalini Ranjan, Barbara Falkensammer, Wegene Borena, Michael Meyer-Hermann, Dorothee von Laer

**Affiliations:** 1 Institute of Virology, Department of Hygiene, Microbiology and Public Health, Medical University of Innsbruck, Innsbruck, Austria; 2 Department of Systems Immunology and Braunschweig Integrated Centre of Systems Biology, Helmholtz Centre for Infection Research, Braunschweig, Germany; 3 Dr. Walser’s surgery, Ischgl, Austria; 4 Department of Medical Statistics, Informatics and Health Economics, Medical University of Innsbruck, Innsbruck, Austria; 5 Centre for Mind/Brain Sciences, University of Trento, Trento, Italy; 6 Institute for Biochemistry, Biotechnology and Bioinformatics, Technische Universität Braunschweig, Braunschweig, Germany

**Keywords:** SARS virus, Viral epidemiology, Viral infection

## Abstract

**Background:**

In early March 2020, a SARS-CoV-2 outbreak in the ski resort Ischgl in Austria initiated the spread of SARS-CoV-2 throughout Austria and Northern Europe.

**Methods:**

Between April 21^st^ and 27^th^ 2020, a cross-sectional epidemiologic study targeting the full population of Ischgl (*n* = 1867), of which 79% could be included (*n* = 1473, incl. 214 children), was performed. For each individual, the study involved a SARS-CoV-2 PCR, antibody testing and structured questionnaires. A mathematical model was used to help understand the influence of the determined seroprevalence on virus transmission.

**Results:**

The seroprevalence was 42.4% (95% confidence interval (CI) 39.8–44.7). Individuals under 18 showed a significantly lower seroprevalence of 27.1% (95% CI 21.3-33.6) than adults (45%; 95% CI 42.2–47.7; OR of 0.455, 95% CI 0.356–0.682, *p* < 0.001). Of the seropositive individuals, 83.7% had not been diagnosed to have had SARS-CoV-2 infection previously. The clinical course was generally mild. Over the previous two months, two COVID-19-related deaths had been recorded, corresponding to an infection fatality rate of 0.25% (95% CI 0.03–0.91). Only 8 (0.5 %) individuals were newly diagnosed to be infected with SARS-CoV-2 during this study.

**Conclusions:**

Ischgl was hit early and hard by SARS-CoV-2 leading to a high local seroprevalence of 42.4%, which was lower in individuals below the age of 18 than in adults. Mathematical modeling suggests that a drastic decline of newly infected individuals in Ischgl by the end of April occurred due to the dual impact from the non-pharmacological interventions and a high immunization of the Ischgl population.

## Plain Language Summary

The Austrian ski resort of Ischgl was a major hot-spot of SARS-CoV-2 infection early on in the pandemic. Here, we investigated the number of people that had been previously infected with the virus in Ischgl in April 2020, by measuring antibodies, blood proteins produced in response to the virus, against SARS-CoV-2. We found that these antibodies were present in 42.4% of the individuals tested and that the majority of these infections, which were generally mild, had not been previously detected. Children were less likely to have these antibodies compared to adults. Using mathematical models, we also found that the subsequent decline in SARS-CoV-2 infections was likely caused by a combination of immunity in the population of Ischgl and of non-pharmaceutical interventions. Our findings allow a better understanding of localised outbreaks of SARS-CoV-2 and how these might be controlled in future.

## Abstract (web summary)

Knabl et al. perform a seroepidemiological study in the Austrian ski resort Ischgl, where a super-spreader event lead to a SARS-CoV-2 outbreak. Through mathematical modelling, they find that the subsequent decline in viral transmission was most likely a combined effect of high seropositivity and the implementation of non-pharmaceutical interventions.

## Introduction

Since December 2019, the severe acute respiratory syndrome coronavirus 2 (SARS-CoV-2) has spread from Wuhan, China, to all continents causing a severe pandemic^1–4^. Globally, as of 7 February 2021, there were over 105 million confirmed cases of COVID-19, including over 2.3 million deaths, reported to the WHO^5^. In Austria, the first cases were detected on 25 February 2020 and until early March, only few limited outbreaks were observed. Then a super-spreading event occurred in an après-ski bar in the ski resort Ischgl leading to an explosive local outbreak associated with the spread of the virus throughout Austria and to many other European countries and worldwide^6,7^. Since then, epidemiological cluster analyses performed by the Austrian Agency for Health and Food Safety (AGES) found that by 20 April up to 40% of SARS-CoV-2 infections in Austria could be traced back to the epicenter Ischgl^8^.

The bar was closed on 8 March and all après-ski bars in Ischgl on 10 March. After the tourists and most guest workers had left, the valley, in which Ischgl lies, was quarantined on 13 March and remained completely isolated for 6 weeks before our study started. In this isolated population, we determine sero- and virus positivity, model the effect of the high seropositivity on virus transmission and analyze the clinical course, hospitalization and fatality rate, and percentage of non-diagnosed cases. The results of our study show that a high seroprevalence of 42.4% can help to control virus spread when some mild non-pharmaceutical interventions (NPIs) are in place.

## Methods

### Study population, study design, and recruitment

The ethical committee of the Medical University of Innsbruck approved the study (EC numbers: 1100/2020 and 1111/2020), which took place between 21 and 27 April 2020. All relevant ethical regulations were followed and informed consent was obtained from all participants. This cross-sectional epidemiological survey targeted all residents of Ischgl/Tyrol irrespective of age and gender. At the time of investigation, Ischgl had a population size of 1867 individuals—1617 with their main residence in Ischgl and 250 seasonal immigrant workers—living in 582 different households^9^. The number of seasonal workers is a value estimated by the local authorities. Most of the over 2000 workers present in Ischgl in early March left before or directly after the quarantine was initiated without signing out. All households in Ischgl were contacted by a postal invitation to attend the study center at a specific time for each household, whereby 1534 individuals from 478 households participated, corresponding to a participation rate of 82.7% and 63.5% of the adult and pediatric population, respectively (Supplementary Fig. 1). As the study was anonymous and some individuals did not visit the study center together with the rest of the household members, 106 individuals could not be assigned to a household. After exclusion of 61 study participants (due to missing of one of the biosamples, i.e., blood sampling was not possible in 2 children and 59 adults refused swab sampling), 1473 (79% of the Ischgl population) were included in our statistical analysis. Structured questionnaires were used to collect data on age, household affiliation, the occurrence of physical symptoms after 1 February 2020, and previous SARS-CoV-2 PCR results. The basic characteristics of the study participants are given in Table 1. The participants were screened for anti-SARS-CoV-2 antibodies as well as for SARS-CoV-2 virus in nasopharyngeal swabs by three immunoassays for binding antibodies, a neutralizing antibody assay (nAb-assay) and an RT-PCR, respectively.

**Table 1:** Baseline characteristics of study participants.

A. Sociodemographic data		
Variables	Value	
*Number of participants, n (%)*	1473 (100)	
Male	712 (48.3)	
Female	759 (51.5)	
Diverse	2 (0.1)	
*Age (years), mean (SD), median*		
Male	40.3 (19.5) 40	
Female	40.9 (20.1) 40	
Children (<18)	10.2 (4.9) 10.5	
Adults (≥18)	45.8 (16.5) 45	
*Age (years) distribution, n (%)*		
<6	52 (3.5)	
6–9	49 (3.3)	
10–13	57 (3.9)	
14–17	56 (3.8)	
18–24	124 (8.4)	
25–34	256 (17.4)	
35–44	239 (16.2)	
45–54	238 (16.2)	
55–64	225 (15.3)	
65–74	117 (7.9)	
≥75	60 (4.1)	

B. Clinical and laboratory data		
Variable	Adults	Children
*Reported hospitalization due to SARS-CoV-2, n (%)*		
Male	7 (1.2)	0
Female	2 (0.3)	0
Diverse	0	n.a.
*PCR*_*self-reported*_ *positive, n (%)*		
Male	45 (7.4)	2 (1.9)
Female	54 (8.3)	4 (3.6)
Diverse	0	n.a.
*PCR*_*tested at study site*_ *positive*^*a*^*, n (%)*		
Male	4 (0.7)	2 (1.9)
Female	2 (0.3)	1 (0.9)
Diverse	0	n.a.
*Seroprevalence*_*(2 of 4 antibody tests positive)*_*, n (%)*		
Male	284 (46.7)	26 (25.0)
Female	281 (43.3)	32 (29.1)
Diverse	1 (50)	n.a.

^a^One of the nine study-site-PCR positive participants was tested SARS-CoV-2-PCR positive before (on 23.03.2020).

### SARS-CoV-2-PCR, anti-SARS-CoV-2- antibody tests

RT-PCR for the detection of SARS-CoV-2 was performed using the RealStar® SARS-CoV-2 RT-PCR kit 1.0 (Altona Diagnostics GmbH, Hamburg, Germany). Ct values below 40 were rated as positive and confirmed by a second PCR. The district administration provided results of SARS-CoV-2 PCR tests in Ischgl, which had been performed between March 1 and April 20, 2020 by different laboratories using commercially available and validated kits.

Participants’ sera were screened for anti-SARS-CoV-2-S1-protein IgA and IgG positivity by a commercially available anti-SARS-CoV-2-IgA and -IgG ELISA (Euroimmun, Lübeck, Germany), respectively, and for anti-SARS-CoV-2-N-protein IgG (anti-N IgG) with the Abbott SARS-CoV-2 IgG immunoassay (Abbott, Illinois, USA). Borderline values in the Euroimmun IgG ELISA were rated positive.

### Neutralizing antibody-assay

Neutralizing antibody (nAb)-assays using highly SARS-CoV-2 susceptible cells^10^ were performed as described in detail in the supplement. We tested all 50 anti-spike protein (S) IgG+/anti-nucleoprotein (N) IgG+ positive children, 148 randomly selected currently PCR negative adults that were positive for anti-S IgG as well as anti-N-IgG, all PCR negative subjects with discrepant antibody results (*n* = 38 for anti-S IgG+/anti-N IgG- and *n* = 36 for anti-S IgG−/anti-N IgG+) or with solely anti-S IgA positive ELISA results (*n* = 26). In addition, all nine currently PCR positive patients were analyzed (Table 2). Plasma samples from 10 blood donors from early 2019 served as negative controls.

**Table 2:** Determining true antibody positivity.

	Anti-S IgG + Anti-N IgG +	Anti-S IgG + Anti-N IgG-	Anti-S IgG - Anti-N IgG +	Anti-S IgA only^c^	3 ELISAs negative and PCR positive^d^	3 ELISAs negative and PCR negative
Total *N* = 1473 (%)	544 (36.9)	38 (2.6)	36 (2.4)	26 (1.7)	6 (0.4)	823 (55.9)
Neutralisation Assay >1:4	201/201^a^ (100)	32/37^b^ (88.5)	36/36 (100)	10/26 (38.5)	1/3 (33.3)	n.a.
Rate as positive *n* = 624 (%)	544 (87.2)	34 (5.4)	36 (5.8)	10 (1.6)	0 (0.0)	0 (0.0)
Reported PCR positive *n* = 105 (%)	95 (90.5)	3 (2.9)	4 (3.8)	0 (0.0)	3 (2.9)	0 (0.0)
New PCR-confirmed cases *n* = 8^e^ (%)	2 (25)	1 (12.5)	0 (0.0)	2 (25)	3 (37.5)	0 (0.0)

S spike protein, *N* nucleocapsid protein, *n.a.* not applicable.

^a^Not all Anti-S IgG+ Anti-N IgG+ positive samples were tested in the neutralization assay.

^b^No sample left for neutralizing antibody assay for one patient.

^c^Anti-S IgA available for *n* = 975 participants.

^d^PCR positive includes results of self-reported PCR prior to study as well as positive PCR results obtained during the study.

^e^One person excluded as PCR positivity already known (included in the reported PCR positive group).

As the immunoassays can produce false-positive results, they are generally confirmed by a neutralizing antibody assay. However, out of 400 sera from 2019, no serum was found to be positive in both immunoassays used here (manuscript in preparation). Thus, the combination of both antibody assays, requiring both to be positive for a final positive result, had a specificity of 100%. Therefore, all sera were tested in two different antibody assays and samples that were positive in both anti-S IgG and anti-N-IgG antibody assays were not all retested by the neutralizing antibody assay. To maximize sensitivity, all antibody discrepant sera were tested in the neutralizing antibody assay. Thereby a sensitivity of nearly 100% for the presence of antibodies to SARS-CoV-2 is expected to be achieved. Reported performances of the antibody assays used have been described in detail previously^11^.

For the maximally four different methods for anti-SARS-CoV-2 antibody detection, the following finding constellations were classified as seropositive:

(I) Positive result in anti-S1-protein IgG ELISA AND anti-N-protein IgG immunoassay.
(II) Positive result in anti-S1-protein IgG ELISA OR anti-N-protein IgG immunoassay and a positive result in the nAb-assay.
(III) Positive result in anti-S1-protein IgA ELISA AND positive result in nAb-assay.

### Statistical and mathematical analysis

Demographic characteristics were tabulated using descriptive statistics including the calculation of means ± standard deviations for continuous measures and numbers (%) for categorical measures. 95% confidence intervals (CIs) for crude prevalence estimates (self-reported PCR testing, seroprevalence, hospitalization, and infection fatality rate), overall and stratified by sex and adults/children, were computed by the method of Clopper and Pearson^12^. General estimating equation (GEE) models according to Zeger and Liang were employed to model associations of self-reported symptoms with seropositivity outcomes, taking into account the possible interdependencies of study participants living in the same household^13^. The age- and sex-adjusted GEE models were specified with a logistic link function, an exchangeable correlation structure, and robust standard errors to estimate odds ratios (OR) and 95% CI. To prevent confounding by influenza-like symptoms during its peak season and considering the lack of firm evidence of COVID-19 cases in Ischgl until the third week of February, self-reported symptoms occurring during peak influenza season (before 23 February) were excluded from the GEE analysis^14^. However, the existence of COVID-19 cases in Ischgl during the last week of February 2020 can be deducted from the fact that 11 individuals out of the first 14 Islandic detected COVID-19 cases infected in Ischgl returned to Iceland on 29 February 2020^7^.

A household analysis of all households was performed as described in detail in the supplement.

The SARS-CoV-2 outbreak in Ischgl was modeled using an age-unstratified compartmental model of infection epidemics based on ordinary differential equations (ODEs), specifically adapted to the biological features and clinical course associated with SARS-CoV-2 infection (see Supplementary Methods). Ischgl being a ski resort with tourist turn-over and a relatively small number of indigenous residents is expected to have a distinct local contact matrix than that of the national average of Austria. In addition, the absence of data regarding the changes of contact frequency among different age groups due to NPIs makes data-based parametrization of an altered contact matrix during the imposition of the NPIs almost impossible for Ischgl. While these limitations forbid us from using an age-stratified mathematical model based on contact matrix^15,16^ for the current study, our goal to infer an overall infection transmission dynamics in Ischgl, future projections and seropositivity remain undisturbed. All underlying datasets and codes describing the model are available online^17^.

### Reporting Summary

Further information on research design is available in the Nature Research Reporting Summary linked to this article.

## Results

### Seroprevalence

**Fig. 1:**
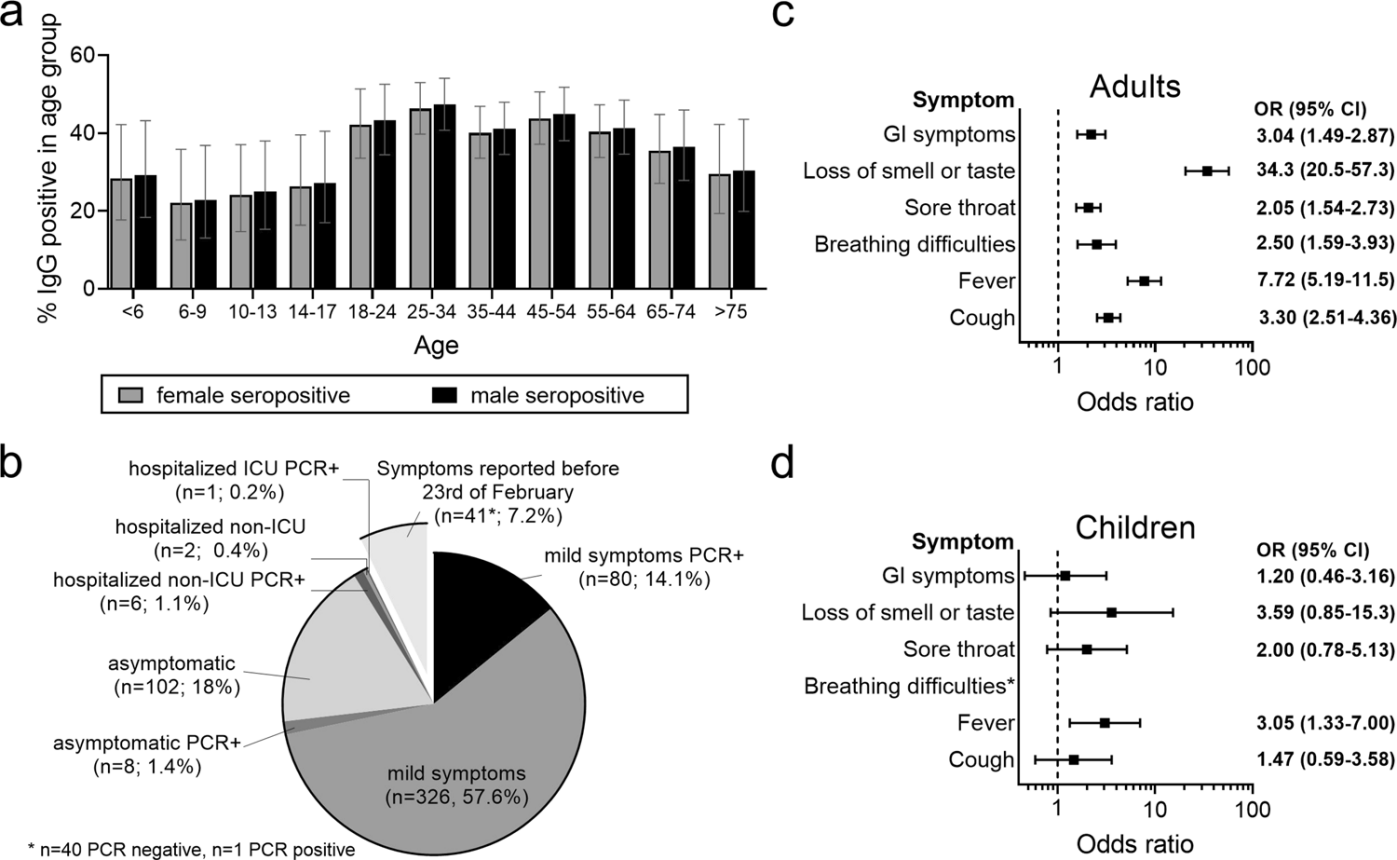
Seroprevalence and clinical course. **a** The seroprevalence in different sex and age groups was calculated using a generalized estimating equation (GEE)-model taking household clusters into account, *n* = 1473. Shown are estimated means and 95% CI. **b** Clinical courses of COVID-19 in seropositive adult individuals (*n* = 566) were classified based on the information provided by the study participants. Mild disease was defined as reported COVID-19-associated symptoms not requiring hospitalization. In addition, all study participants were asked if a SARS-CoV-2 PCR was carried out and its result. Symptoms reported before February 23^rd^ were excluded as the peak of the influenza season was in the first half of February and the odds ratios(OR) for reported symptoms and SARS-CoV-2 seropositivity were low then. **c** Odds ratios of self-reported symptoms regarding seropositivity in adults (*n* = 1259) were calculated using generalized estimating equation (GEE)-model adjusted for age and sex, taking household clustering in account. **d** Odds ratios of self-reported symptoms and symptoms reported by persons of care and custody, respectively, regarding seropositivity in children (*n* = 214) were calculated using generalized estimating equation (GEE)-model adjusted for age and sex, taking household clustering in account). Shown in (**c**) and (**d**) are odds ratios and 95% CI.

From a total of ~1867 people living in Ischgl, 1473 individuals living in 478 households including all age groups were tested for antibodies to SARS-CoV-2 and for SARS-CoV-2 virus RNA in nasopharyngeal swabs (Supplementary Fig. 1). This included 101 children under the age of 10 and 113 participants between 10 and 17 (Table 1). A total of 624 study participants were seropositive in at least two of maximally four antibody tests performed (3 binding antibody assays + neutralizing antibody test) corresponding to a seroprevalence of 42.4% (95% CI 39.8–44.7, Table 2). The IgG values for the anti-SARS-CoV-2 S protein antibodies (Euroimmun ELISA) correlated well with the anti-N IgG levels (Abbott immunoassay) (Supplementary Fig. 2, *R* = 0.8). All sera that were positive in only one of the two assays were tested for neutralizing antibodies (Table 2, Supplementary Fig. 3). The values in the anti-S protein IgG ELISA correlated well with the neutralizing antibody titers, *R* = 0.6, *p* < 0.001 (Supplementary Fig. 4). The seroprevalence was significantly lower in children and adolescents under 18 than in adults, with 27.1% (95% CI (21.0–33.6)) and 45% (95% CI (42.2–47.7)), respectively (OR of 0.455, 95% CI 0.356–0.682, *p* < 0.001; Fig. 1). The seroprevalence in women 41.2% (37.5–44.9) did not differ significantly from men 43.5% (39.5–47.3) (OR 0.91 (0.74–1.12), *p* = 0.37; Fig. 1a).

### Prevalence of virus positivity in PCR test

**Fig. 2:**
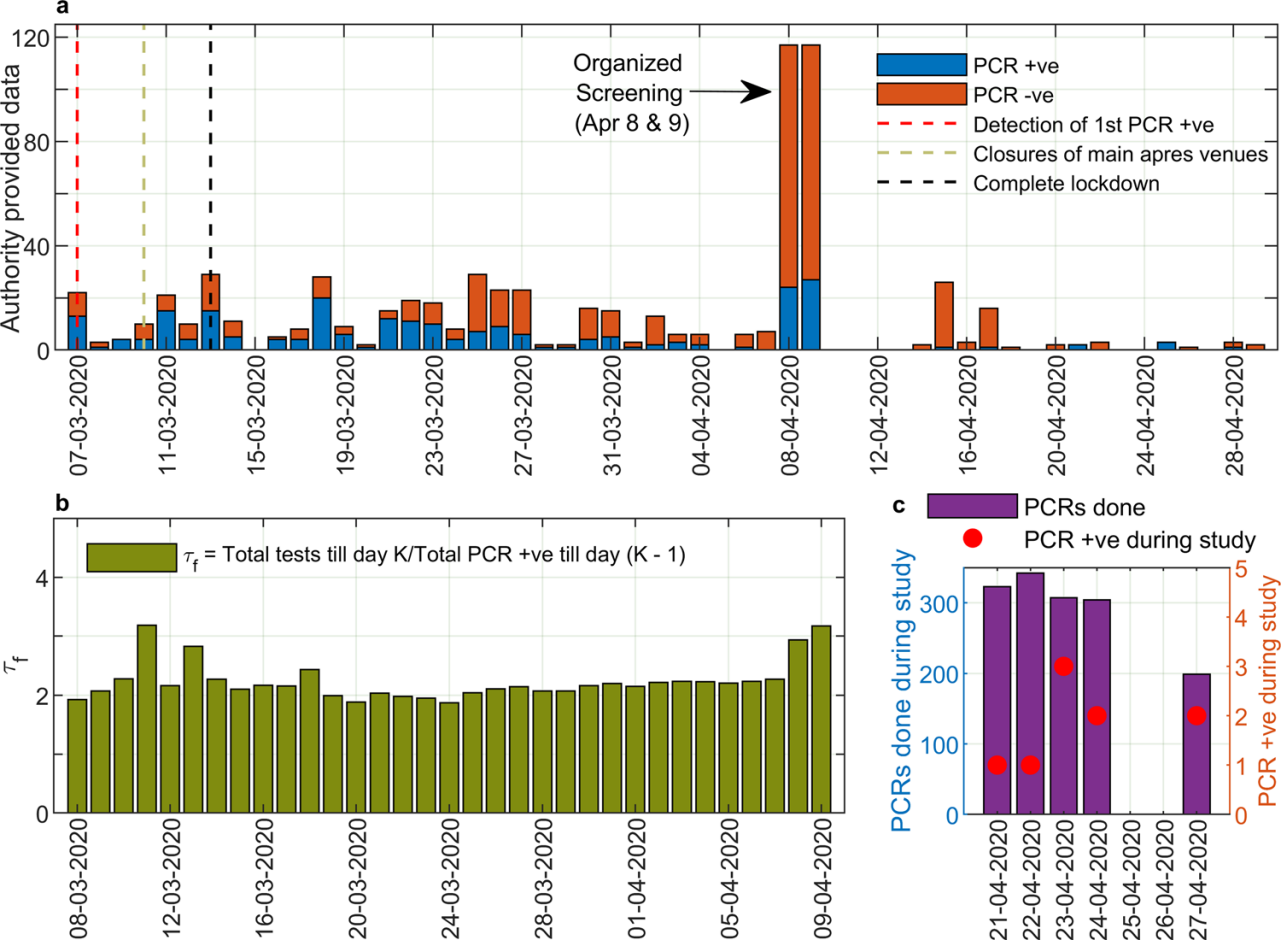
Analysis of PCR testing in Ischgl. **a** SARS-CoV-2 PCR results from 7 March to 29 April 2020 were provided by the district administration. On 7 March 2020 the first person in Ischgl was tested positive for SARS-CoV-2. On 10 March 2020, most of the main entertainment venues in the ski resort were closed. On 13 March 2020, a complete lockdown was declared due to a rapidly increasing number of new cases and the village was quarantined. On 8 April and 9 April 2020, the state health authority carried out a screening examination in which 234 people were tested for SARS-CoV-2. **b** From the PCR testing data provided by the local authority, we calculated the average number of tests done per confirmed PCR result (${\tau }_{f}$), in terms of the ratio of total PCR tests done till a day and total number of PCR positive individuals till one day prior to it. **c** The PCR screening during this study was performed between 21 April and 27 April 2020 in Ischgl.

In 9 of the 1473 (prevalence 0.6%, 95% CI (0.3–1.2)) study participants, viral RNA was found in nasopharyngeal swabs by PCR (Fig. 2, Supplementary Table 1). In all cases, the level of viral RNA was low and all individuals presented without symptoms (Supplementary Table 1). Of these, eight cases were new. The already known PCR positive case had been tested more than a month positive prior to our study, which has also been previously been described by others^18^. Five of the eight new cases were positive for neutralizing antibodies. Four PCR positive individuals reported to have had symptoms up to 39 days before the study of which three had the typical loss of smell and taste (Supplementary Table 1). One individual had PCR-confirmed COVID-19 39 days before the study begins. This shows that viral RNA can be detected for longer periods even if anti-viral neutralizing antibodies are already present.

Only 105 participants reported to have been diagnosed with SARS-CoV-2 infection by PCR previously. Of these, 102 were seropositive (Table 2), showing that almost (97%) all previously PCR+ cases had seroconverted by the time the study was initiated. Thus only 102 of the 624 past SARS-CoV-2 infections diagnosed in this study by serology were previously PCR+ and reported to the authorities (16.3%, 95% CI 13.5–19.5), corresponding to 83.7% non-diagnosed SARS-CoV-2 infections in a known hot spot considered to be the epicenter in Austria. The proportion of PCR-diagnosed and reported cases was lower in children and adolescents under the age of 18 (10.3%, 95% CI 3.9–21.2) than in adults (17.0%, 95% CI 13.9–20.3; Supplementary Fig. 5).

In Fig. 2a, the results of the PCRs testing coordinated by the local authorities are given. Also, here the highest percentage of positive PCR was seen mid-March, when the highest number of participants reported to have had clinical disease. As per the data in Fig. 2a, the general trend for the average number of PCR tests done per confirmed PCR result (${\tau }_{f}$) as defined by the ratio of total PCR tests done till a day and a total number of PCR positive individuals till one day earlier, remained low in Ischgl with a value of 2–3 (Fig. 2b).

### Clinical course

For the clinical symptoms, we focused on those reported after 23 February because symptoms reported earlier were less significantly linked to SARS-CoV-2 seropositivity, most likely because the peak of the influenza season was in the first half of February and the level of SARS-CoV-2 transmission was still low then. The clinical course as reported by the study participants was mild, defined as not requiring hospitalization, or even asymptomatic in most study participants, with only 9 adults (no children) reporting hospitalization (0.6%, 95% CI (0.3–1.2)) of which only one adult was in the ICU (Fig. 1b). In adults, the major previous symptoms remembered by seropositive participants were typical for mild forms of COVID-19. Loss of smell and taste highly correlated with IgG positivity (OR 34.3), as did fever (OR 7.7) and cough (OR 3.3, Fig. 1c). In contrast, in the much less clinically affected children, reported previous symptoms were less significantly associated with seropositivity (Fig. 1d).

Two patients in the Ischgl population died of COVID-19. The deaths both occurred after 20 March. Relative to the number of seropositive individuals detected in our study, the infection fatality rate (IFR) would be 0.34%. If corrected to the number of seropositive individuals estimated to be living in Ischgl (42.4% of 1867), the IFR in Ischgl was as low as 0.25%, albeit with a broad 95% CI of 0.03–0.91. However, with only two deaths, this IFR in Ischgl is not statistically robust.

### Analysis of household clustering

Among 478 households, there were 184 households where all members were negative (38.5%). In 107 households, all members were positive (22.4%) and 187 households were of mixed status (39.1%). Among all 478 households, 124 households contained children. Among these households *n* = 84 households (67.7%) did not have any positive children, even though *n* = 51 of these households (60.7%) had at least one positive adult. In contrast, there was only one household among the 124 households with children where a child was positive, but no adult was positive. Mixed status households are much more common among households with children than without (Supplementary Table 2). In households with children, the odds of a child of being positive were 66% lower than for adults (OR of 0.44 (95% CI 0.31–0.64).

Due to the participation rate of 79%, there may be incomplete households in this household level analysis. Thus, the estimate of the proportion of households with mixed status regarding seropositivity is conservative. In addition, there were 106 participants with unknown household information. These were excluded from the household analysis.

### Mathematical model of Ischgl outbreak

**Fig. 3:**
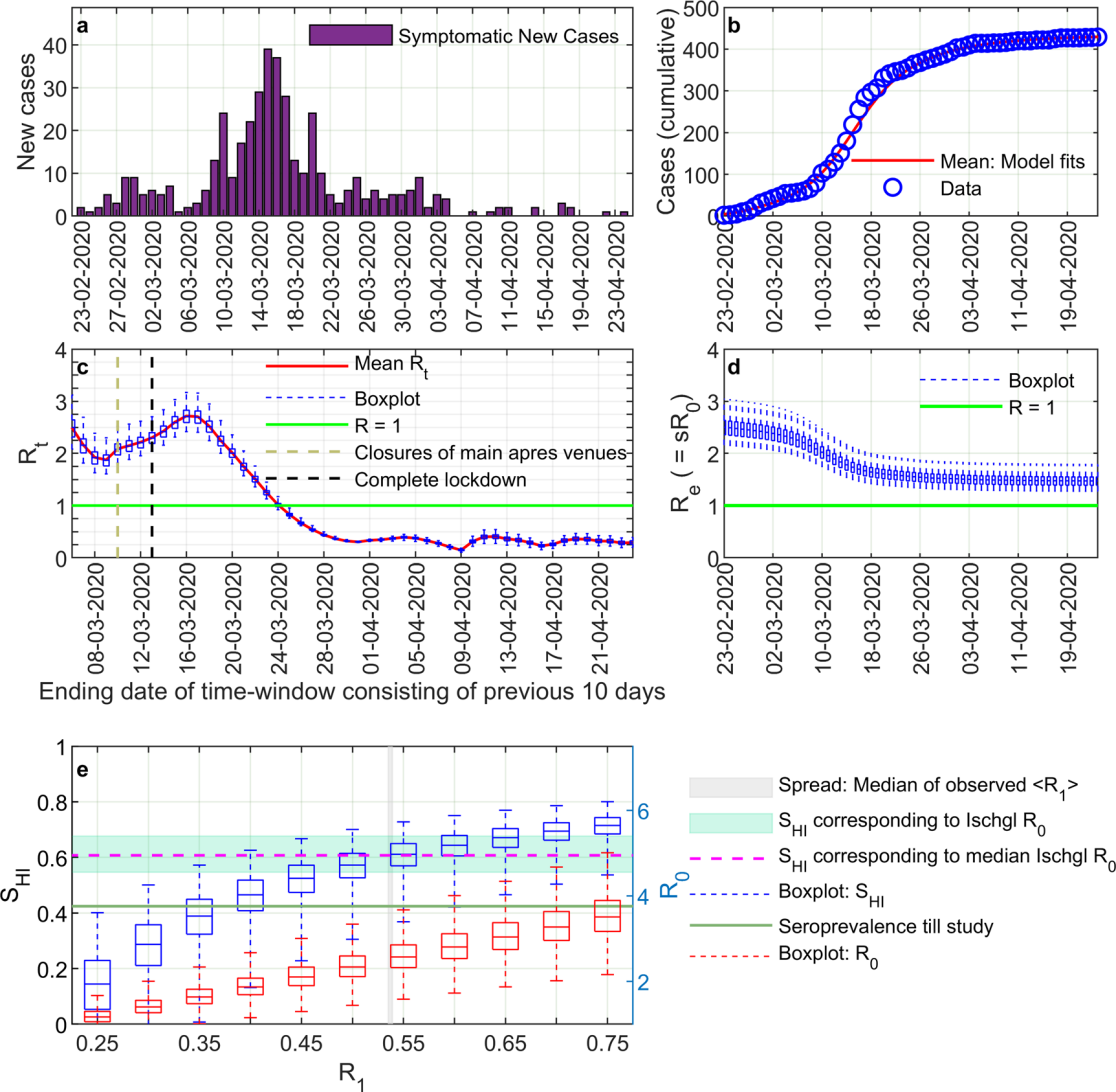
Mathematical analysis of the outbreak. The time-course of new cases in Ischgl based on any COVID-19 associated symptom among seropositive individuals as obtained from the survey (**a**) was used to determine the daily infection transmission rate ($${R}_{1}$$) for each of the sampled parameter sets (see Supplementary Methods). The, thus, calibrated model reproduces the cumulative number of symptomatic cases (**b**) and characterizes the time-dependent reproduction number $${R}_{t}$$ (**c**). Time-dependent alterations in the effective reproduction number $${R}_{e}$$ (**d**) were calculated as $${R}_{e}(t)=s\left(t\right){R}_{0}$$, $$s(t)$$ being the fraction of the susceptible population at a certain time *t*. Seroprevalence necessary for herd immunity ($${S}_{{HI}}$$, green shaded region) and basic reproduction number ($${R}_{0}$$, right ordinate axis, red box plots) are shown as functions of $${R}_{1}$$ (**e**). The analysis was done with 100 parameter sets following calibration of $${R}_{1}$$ to best describe the case numbers until Mar 16 at a full stretch (see fitting strategy in the Supplementary Methods). $${R}_{0}$$ in Ischgl was in between 2.2 and 3.1, with a median value of around 2.5, which corresponds to a median value of around 60% seroprevalence to reach herd immunity ($${S}_{{HI}}$$ corresponding to median $${R}_{0}$$, purple line). This is consistent with herd-immunity seroprevalence (blue box plot) derived from the medians of the mean contact dependent daily transmission rate $$< {R}_{1} > $$ for the time-windows spanning 23 February–16 March and 28 February–16 March (gray patch, bounded by these medians). The observed seroprevalence as of April 2020 (green line) is lower than the thresholds. We assume that the seropositive individuals who reported their onset of symptoms before 23 February 2020 during the survey were not symptomatic due to COVID-19 at that time, rather they might have an asymptomatic course of COVID-19 later. Boxplots show median, interquartile range and whiskers with maximal 1.5× interquartile range.

Next, we addressed with a mathematical model whether the drop in the number of new reported cases during March was solely due to the NPIs or whether Ischgl was on the verge of achieving herd immunity. We also use the epidemiological model to explicitly infer the infection transmission dynamics and temporal variations of different compartments (e.g., susceptible) over the course of the epidemic as well as to project the future course of the outbreak under different circumstances, thereby analyzing beyond the scope of the traditional approaches used to determine the evolution of reproduction numbers from symptom onset data^19^. Simulation results of our computational model (Supplementary Figs. 6 and 7) fitted well with the epidemic curve constructed with the temporal data for onset of any of the COVID-19 symptoms (Fig. 3) and that for the onset of anosmia/dysgeusia among the seropositive individuals (Supplementary Fig. 8) in Ischgl. As most of the residents in Ischgl were engaged in tourism related activities, one of our major assumptions was the homogeneous mixing among the residents and tourists. The basic reproduction number $${R}_{0}$$, which determines the evolution of an epidemic in a non-intervened scenario without external alterations in a local system, is meaningful at the initial phase of an outbreak before NPIs are introduced or behavior of the residents has changed. Based on survey data for onset of any of the COVID-19 associated symptoms from 23 February 2020 (Fig. 3a), we found $${R}_{0}$$ in Ischgl to be between 2.2 and 3.1, with a median value of 2.5 (Fig. 3c). This corresponds to a median value of 60% seroprevalence to reach herd immunity, which is consistent with herd-immunity seroprevalence calculated using a mean contact dependent daily transmission rate $$< {R}_{1} > $$ for the time-windows spanning 23 February–16 March and 28 February–16 March (Fig. 3e).

To determine the temporal variation observed during the whole course of the outbreak (Fig. 3), the time-dependent reproduction number was determined. We opted for a shifting time window of 10 days, in each of which *R*_t_ was determined. The next time window shifted by one day contained the history of the epidemic from the prior time window. The initial condition for the next time window is determined from the saved state of the simulation at the beginning of the next time window as obtained from the optimization of its prior time window^20^. (see Supplementary Methods, Supplementary Figs. 6–15, Supplementary Tables 3–4). Ischgl faced a major outbreak till mid-March (Fig. 3c). The viral spreading infected a substantial fraction of the population between 2 March and 18 March 2020, as reflected by the drop in the effective reproduction number ${R}_{e}$ in Fig. 3d. As ${R}_{e}$ > 1, this implies that if all measures were lifted, a new local outbreak would be expected (Supplementary Figs. 10, 13). The median value of ${R}_{t}$ was already <1 for the time-window ending on 25 March and afterwards (Fig. 3c). This suggests the effectiveness of the NPIs in Ischgl, which was also reflected in the projected course of the outbreak (Supplementary Fig. 11) and with different levels of reduced transmission probabilities during the period of lock-down (Supplementary Fig. 9). The same analysis based on data for anosmia/dysgeusia onset gave qualitatively similar results with a slightly higher ${R}_{0}$ (range: 2.4–3.1, median: 2.6) and, hence, even stronger requirements (median seroprevalence of 61.5%) to reach herd immunity (Supplementary Figs. 8, 12–14).

## Discussion

The high seroprevalence of 42.4% (95% CI 39.8–44.7) observed in Ischgl was based on the combination of four serological tests, two IgG immunoassays, one IgA ELISA, and a neutralizing antibody assay. The empirically measured 42.4% seroprevalence is to our knowledge one of the highest seroprevalences reported so far^21–25^. In New-York City a higher seroprevalence was published, but this was an analysis in patients and not the general population^26,27^. In a study of the Austrian Health Ministry performed by one of Austria’s leading social research institutes (SORA) in the 27 COVID-19 hotspots in Austria outside of Tyrol, 4.6% of 269 individuals tested were seropositive^28^. Thus, the high seroprevalence found here is in accordance with the claim that Ischgl was the epicenter of the COVID-19 pandemic in Austria and central to the spread of SARS-CoV-2 throughout Europe.

The percentage of seropositive children and adolescents under 18 (27%) was significantly lower than the percentage found in adults (45%) (OR 0.455, 95% CI 0.330–0.628, *p* < 0.001). This is in line with studies in Iceland^7^, Toulouse^29^, Geneva^21^, and Wuhan/Shanghai^30^, but not with other studies, where children were found to be infected as frequently as adults^25,31^. While the study in Wuhan/Shanghai concluded that children are less susceptible to SARS-CoV-2 infection due to a biologic/immunological relative resistance to infection, the somewhat abrupt increase in the seroprevalence for individuals over 18 years of age found in Ischgl would argue that here the children were less exposed to the virus than the working adults. If a lower biologic susceptibility of children to SARS-CoV-2 was the only reason, the seroprevalence would be expected to already rise at younger ages and not abruptly at the age of 18, when most of the local population starts to work in local tourism. Nearly 10,000 new tourists arrived each week in addition to several 100 guests each night that come to Ischgl from other ski resorts to visit the apres-ski bars. In conclusion, the higher seroprevalence in the working population >18 relative to children and youths is most likely at least partially explained by a higher exposure.

Of the seropositive individuals, 83.7% had not been diagnosed to have had SARS-CoV-2 infection previously. This percentage is especially high in children and asymptomatic adults and explains the less drastic difference between children and adults in our seroepidemiologic study relative to the reported PCR positive cases in Ischgl (Supplementary Fig. 5). The high level of unreported cases also explains the relatively low IFR of 0.25%, relative to the case fatality rate reported for Austria of 3.7%. Admittedly, the 0.25 % (0.03–0.91) IFR is not a statistically very robust number as it is based on two fatalities only. However, this low IFR is in line with a German seroepidemiologic study with an IFR of 0.37%^25^, which reported similar low numbers of fatalities, and a summary study using data from 12 seroprevalence studies (IFR 0.03–0.5%)^32^ and a study in New York City (IFR 0.97%)^27^ but lower than predicted in a statistical model, 0.5–1.3%^32^. This study includes all infections and not only the reported cases. Thus, as expected, the hospitalization rate of the seropositive study participants of 1.5% was much lower than the hospitalization rates reported for Austria, which are around 15% according to official data from the Austrian registry for mandatorily reportable diseases. The low IFR and hospitalization rate could also partially by the lower seroprevalence in elderly patients. Finally, the high rate of unreported cases suggests that in the future more testing especially in hotspots should be performed to allow a more efficient containment of virus spread.

The prevalence of newly PCR positive individuals was surprisingly low and had dropped from 19% detected in a voluntary public screening of 234 individuals in Ischgl in the first week of April to 0.5% during our study 3 weeks later (Fig. 2a, c). This level is still higher than the 0.15% percent which dropped from a level of 0.33% determined in the rest of Austria during the same period in a governmental surveillance study^28,33,34^.

The interesting question is now whether the achieved virus control in Ischgl is stable. Based on the basic reproduction number ${R}_{0}$ between 2.2 and 3.1, around 60% seropositivity is required to stop spread of SARS-CoV-2 in Ischgl. Fitting of model parameters to the time course of case numbers in Ischgl revealed that the number of seropositive individuals in Ischgl is still quite lower than the herd immunity threshold, thus, suggesting that the installed non-pharmaceutical measures contribute to the containment of the virus. Likely, a release of all measures leading to transmission at the same level as during the touristic season early March, would re-initiate viral spreading. Although a comparison of stochastic and ODE simulations of the model with the same population size as of Ischgl establishes the robustness of our approach even for a low population size, the analysis does not allow for a precise quantitative evaluation of the herd-immunity threshold because of several limitations in the data. Subjective reporting of symptom onset dates as well as an unclear attribution of symptoms to SARS-CoV-2 infections are major sources of uncertainty. It is not known whether a fraction of the population, in particular, of the children, might combat the viral infection by innate immune responses alone without developing specific antibodies, thus, appearing seronegative in the current study. Further, the prevalence of super-spreading-events in Ischgl might impact on the mathematical analysis. Beyond our assumption of homogeneous mixing of the Ischgl residents and tourists, the precise impact of the in- and out-flow of tourists, who participated in infection chains and then left following their stay in Ischgl as well as during the exodus around 13 March 2020, on the mathematical analysis cannot be assessed properly in the absence of data on the serostatus of the tourists. While the relative impact of leaving tourists and NPIs are difficult to disentangle, the drastic decline in the time-dependent reproduction number can be explained by the combination of both together with a high immunization of the Ischgl population. The high seroprevalence alone does not explain the successful limitation of virus transmission. Population heterogeneity in terms of inherent innate and T-cell immunity, however, might further reduce the herd immunity threshold^35,36^.

One major weakness of the study is the data set generated from the structured questionnaires leading to a recall bias of PCR tests, of previous symptoms and of the associated time points. In addition, the mathematical model has several limitations that are clearly described above. Furthermore, in the absence of sufficient information on the ‘local’ contact matrix of the ski resort, age-specific disease susceptibility and heterogeneous impact of NPIs on different age groups (e.g., children vs adults) of Ischgl, an explicit age-structured study using the mathematical model stands beyond the scope of the present study. The strengths of the study are, however, that 79% of the population in Ischgl participated including 214 children, that a very extensive antibody testing was performed that gave highly reliable results, and that one of the highest so far reported seroprevalences in a hotspot was found, which was lower in children than in adults and that was associated with a drastic decline in new infections. As herd immunity was most likely not reached as of April 2020, continuation of NPIs appeared to be the suitable option to prevent virus circulation in Ischgl. In addition, susceptibility to reinfection, immunity waning rate, the appearance of new virus variants with altered features, and vaccinations might play a role in determining the course of the outbreak in Ischgl in long run.

## Supplementary information

Supplementary Information

Description of Additional Supplementary Files

Supplementary Data 1

Reporting Summary

## Data Availability

Source data for Fig. 1 is available as Supplementary Data and for Figs. 2 and 3 at 10.5281/zenodo.4704076^17^. All other data are available from the corresponding authors upon receipt of a suitable request.

## Code availability

All simulation results and source code for the implementation of the model and related algorithms are freely available at 10.5281/zenodo.4704076.
