# Supplementary material for: High SARS-CoV-2 seroprevalence in children and adults in the Austrian ski resort of Ischgl
Source: Commun Med (Lond). 2021 Jun 30;1:4. doi: 10.1038/s43856-021-00007-1 (PMC8633917; doi:10.1038/s43856-021-00007-1)
Supplement: Supplementary file 1 — Supplementary Information [file 43856_2021_7_MOESM1_ESM.docx]

**Supplementary Information**

**High SARS-CoV-2 seroprevalence in children and adults in the Austrian ski resort of Ischgl**

Ludwig Knabl^1,^*, Tanmay Mitra^2,^*, Janine Kimpel^1,^*, Annika Rössler^1^, André Volland^1^, Andreas Walser^3^, Hanno Ulmer^4^, Lisa Pipperger^1^, Sebastian C. Binder^2^, Lydia Riepler^1^, Katie Bates^4^, Arnab Bandyopadhyay^2^, Marta Schips^2^, Mrinalini Ranjan^2,5^, Barbara Falkensammer^1^, Wegene Borena^1,$^, Michael Meyer-Hermann^2,6,$,#^, Dorothee von Laer^1,$,#^

^1^ Institute of Virology, Department of Hygiene, Microbiology and Public Health, Medical University of Innsbruck, Innsbruck, Austria

^2^ Department of Systems Immunology and Braunschweig Integrated Centre of Systems Biology, Helmholtz Centre for Infection Research, Braunschweig, Germany

^3^ Dr. Walser’s surgery, Ischgl, Austria

^4^ Department of Medical Statistics, Informatics and Health Economics, Medical University of Innsbruck, Innsbruck, Austria

^5^ Centre for Mind/Brain Sciences, University of Trento, Trento, Italy

^6^ Institute for Biochemistry, Biotechnology and Bioinformatics, Technische Universität Braunschweig, Braunschweig, Germany

* These authors contributed equally

^$^ These authors jointly supervised this work

^#^Corresponding authors:

Michael Meyer-Hermann, Department of Systems Immunology and Braunschweig Integrated Centre of Systems Biology, Helmholtz Centre for Infection Research, Braunschweig, Germany

E-Mail: mmh@theoretical-biology.de

Dorothee von Laer, Institute of Virology, Medical University of Innsbruck,

Schöpfstraße 41, AT-6020 Innsbruck, Austria, Phone: +43 512 9003 71700,

E-Mail: [dorothee.von-Laer@i-med.ac.at](mailto:dorothee.von-Laer@i-med.ac.at)

**SUPPLEMENTARY METHODS**

*Viral RNA detection from throat swabs*

Viral RNA was extracted from the virus transport media (CDC,  SOP#: DSR-052-01 ) using the EasyMag® NucliSENS® System (bioMérieux, Marcy-l'Étoile, France). RT-PCR for the detection of SARS-CoV-2 was performed using the RealStar® SARS-CoV-2 RT-PCR kit 1.0 (Altona Diagnostics GmbH, Hamburg, Germany) on the CFX96TM Real-Time System (Bio-Rad, Feldkirchen, Germany) according to the manufacturers’ instructions. Ct values below 40 in the PCR were rated as positive and confirmed by a second PCR. The district administration provided results of previous SARS-CoV-2 PCR tests in Ischgl, which had been performed between March 1^st^ and April 20^th^, 2020.

*Viral antibody testing*

Participants’ sera were screened for anti-SARS-CoV-2-S1-protein IgA and IgG positivity by a commercially available anti-SARS-CoV-2-IgA and -IgG ELISA (Euroimmun, Lübeck, Germany), respectively, using the fully automated 4-plate benchtop instrument Immunomat™ (Virion/Serion, Würzburg, Germany). Results with respect to the obtained optical density (OD) values were interpreted according to the recommendations in the manufacturer’s information. Samples with a borderline (0.8-1.1) result were repeatedly tested and considered positive in case of another borderline result. Additionally, each serum was tested for anti-SARS-CoV-2-N-protein IgG (anti-N IgG) with the Abbott SARS-CoV-2 IgG immunoassay on the ARCHITECT i2000SR system (Abbott, Illinois, USA). Anti-N IgG was positive, if the obtained relative light unit (RLU) value corresponded to the manufacturer’s recommendations (>1.4).

For the neutralizing antibody assay, Vero cells expressing TMPRSS2 were kindly provided by Dr. Markus Hoffmann and Prof. Stefan Pöhlmann ^1^. SARS-CoV-2 was isolated from a patients’ respiratory swab sample in Innsbruck (isolate 1.2) and virus stocks were produced on Vero/TMPRSS2 cells. Participants’ plasma was heat inactivated at 56°C for 30 min. Subsequently, it was centrifuged for 5 minutes at 8,000 rpm in a tabletop centrifuge. 4-fold plasma dilutions were mixed with an equal volume of SARS-CoV-2 virus (1.2 isolate) resulting in ~300 infected cells in non-neutralized wells. Plasma/virus mixes were incubated for 1 hour at 37°C and subsequently transferred to 96-wells containing 90% confluent Vero/TMPRSS2 seeded one day before. Cells were infected with the virus for 1 hour at 37°C and subsequently washed once, fresh complete medium containing 2% fetal calf serum (FCS) was added and cells were further cultured for 13 hours. Cells were fixed for 5 minutes with 96% ethanol and subsequently stained using the serum from a SARS-CoV-2 recovered patient and a horse radish peroxidase (HRPO)-conjugated anti-human secondary antibody (Dianova, Hamburg, Germany). Plates were developed using 3-amino-9-ethylcarbazole (AEC) substrate. Infected cells were counted in the microscope and 50% neutralization titers were calculated as highest dilution where mean infection of duplicate samples was lower than 50% of the mean of control wells without serum.

*Household analysis*

Sero-status of households with no, 1 or more than 2 children was analyzed. Individuals with unknown household affiliation (n=106) were excluded from the analysis. Household sero status was defined as "positive" (all members positive), "negative" (all members negative) and "mixed" (members both positive and negative). Descriptive statistics were used to describe the household sero status for the whole population, as well as among households with children. An individual level logistic regression model was run for sero status by children/adults for all participants in households with children.

*Mathematical Model*

**Model equations ^2^:**

$$\frac{dS}{dt} = -\frac{R_{1}}{N}\left[ \gamma\left( C \right._{R}+C_{I} \right)+\beta\left( I_{H}+I_{R} \right)+\omega I'+\chi I_{X}]S$$

$$\frac{dE}{dt} = \frac{R_{1}}{N}\left[ \gamma\left( C \right._{R}+C_{I} \right)+\beta\left( I_{H}+I_{R} \right)+\omega I'+\chi I_{X}]S-R_{2}E$$

$$\frac{dC_{I}}{dt} = {\left( 1-\alpha\right)R}_{2}E-R_{3}C_{I}$$

$$\frac{dC_{R}}{dt} = \alpha R_{2}E -{R_{9}C}_{R}$$

$$\frac{dI'}{dt}= \mu R_{3}C_{I}- \tau I^{'}$$

$$\frac{dI_{X}}{dt}=\left( 1 - \mu\right)R_{3}C_{I}- R_{4}I_{X}$$

$$\frac{dI_{R}}{dt} = \left( 1-\rho\right)\tau I'-R_{4}'I_{R}$$

$$\frac{dI_{H}}{dt} = \rho\tau I'-R_{6}'I_{H}$$

$$\frac{dH_{R}}{dt} = \left( 1-\vartheta\right)R_{6}'I_{H}-{R_{5}H}_{R}$$

$$\frac{dH_{U}}{dt} = \vartheta R_{6}'I_{H}-R_{7}H_{U}$$

$$\frac{dH_{s}}{dt}=R_{8}U_{R}-R_{5}H_{S}$$

$$\frac{dU_{R}}{dt} =\left( 1-\delta\right)R_{7}H_{U}-R_{8}U_{R}$$

$$\frac{dU_{D}}{dt} = \delta R_{7}H_{U}-R_{10}U_{D}$$

$$\frac{dR_{X}}{dt} = {R_{9}C}_{R}+{R_{4}I}_{X}$$

$$\frac{dR_{Z}}{dt} = {R_{4}'I}_{R}+{R_{5}H}_{R}+{R_{5}H}_{S}$$

$$\frac{dD}{dt} = R_{10}U_{D}$$

The set of coupled ordinary differential equations are integrated using the stiff solver ode15s implemented in *MATLAB Release 2018a*.

**Model parameters:**

Parameters with fixed value: $\gamma=1, \omega=1, \chi=1, \mu=1, \tau=2, \vartheta=\frac{3}{11}, \delta=\frac{2}{3}$

For analysis with onset of any COVID-19 associated symptom since February 23^rd^, 2020, $\alpha=0.3147, \rho=0.026$

For analysis with onset of anosmia/dysgeusia since February 23^rd^, 2020, $\alpha=0.5735, \rho=0.0412$

**Equation for** $\boldsymbol{R}_{\boldsymbol{0}}$**:**

$$R_{t}= R_{1}(t)\frac{\left. S(t \right)}{\left. N(t \right)}\left[ \frac{\gamma\alpha}{R_{9}}+ \frac{\left. \gamma(1-\alpha\right)}{R_{3}}+\frac{\chi\left( 1-\alpha\right)\left( 1-\mu\right)}{R_{4}}+\frac{\mu\omega\left( 1-\alpha\right)}{\tau}+ \frac{\beta\mu\left( 1-\alpha\right)\left( 1- \rho\right)}{R_{4}'}+ \frac{\beta\mu\rho\left( 1-\alpha\right)}{R_{6}'} \right]$$

We calculated $R_{0}$ using next generation method ^3-5^.

**Data preparation for modelling:**

In this study, the total number of serologically positive people who reported any symptom was: (624 - 136) + 2 = 490, 136 being the number of seropositive individuals who reported not to have any symptom and 2 being the number of total deaths (assumed to be seropositive before death). 29 seropositive persons had not given any symptom onset date and we did not know the symptom onset date of 2 dead persons. These two deaths in Ischgl occurred on 22^nd^ of March and on 10^th^ of April. From the bounds of the system parameters as mentioned in Table S3, the time to death from symptom onset ($\frac{1}{R_{6}}+ \frac{1}{R_{7}}+ \frac{1}{R_{10}}$ days) can be between 13.5 to 29 days. Therefore, 1 of them was placed randomly in between February 23^rd^ and March 8^th^ to have a symptom onset, and the other was placed in between March 12^th^ and March 27^th^ to have a symptom onset. The 29 people who did not provided any symptom onset information, had been distributed randomly to have a symptom onset at a random date over the whole period for which people in Ischgl reported an onset of a symptom, i.e., the period from 26^th^ of January to 24^th^ of April. These 31 people (29 + 2) had been assumed to also have loss of smell/loss of taste, and hence a similar random distribution of these 31 was done while working with the loss of smell/loss of taste data. When a non-specific time for symptom onset (for example, mid-March) was provided, corresponding cases were randomly distributed over 7 days around the non-specific time-point. For example, if N people answered mid-March, those N people were randomly distributed over a period of March 12^th^ to March 18^th^. In case a month in general was mentioned as a non-specific answer, persons with such answers were randomly distributed over the corresponding month to have a symptom onset. Based on a significant overlap with the influenza-season in Ischgl until 3^rd^ week of February and officially accepted course of the outbreak in Ischgl, we have considered the symptomatic cases until February 22^nd^ to be non-specific for COVID-19 (see main text). Symptoms until February 22^nd^ could not be clearly attributed to COVID-19 infections. Based on sero-positivity such cases were considered as asymptomatic in order to define the fraction of asymptomatic patients in the model. Following the data smoothing procedures mentioned above, there were 61 symptomatic cases before February 23^rd^, which were considered as asymptomatic. Hence, the analysis for any symptom onset consists of 490 – 61 = 429 individuals. In this case, $\alpha$ = (626 – 429)/626 = 0.3147. As 11 people among these went to hospital, $\rho$ = 11/429 = 0.026 in this case. For analysis with onset data of anosmia/dysgeusia, we have considered individuals reporting any other symptom (OR much lower as compared to anosmia/dysgeusia) as asymptomatic seropositive. Furthermore, as cases before February 23^rd^ have been considered to be asymptomatic, after the corrections for 31 people mentioned above as well as for the people with non-specific onset date for anosmia/dysgeusia as per a similar procedure to that of the analysis based on any symptom onset, a number of 267 people were considered to have onset of anosmia/dysgeusia. Hence, in this case, $\alpha$ = (626 – 267)/626 = 0.5735. Now among these 267 people, 11 have gone to the hospital, which gives $\rho$= 11/267 = 0.0412. Among 11 people who went to hospital, 3 had needed ICUs including the two people who died, providing us with $\vartheta=\frac{3}{11}, \delta=\frac{2}{3}$. As we know the serostatus of all the 1473 people (size of the sub-population in our model) in our study and consider the symptom (or anosmia/dysgeusia) onset data for the serologically positive people, no symptomatic person among the seropositive individuals remains undetected ($\mu=1$) in our computational analysis. To invoke a day/night symptom onset pattern, we assumed that there was a delay of 12 hours between actual onset of symptom(s) and the date the symptomatic individuals mentioned to have developed a symptom, resulting in $\tau=2/day$.

**Initial conditions:**

As 2 dead people were considered in the study, the total initial population was 1475. Recognized symptomatic cases ($I_{H}+ I_{R}$) on the first 4 days were used to determine the corresponding detectable infected cases ($N_{i}/[(1- \alpha)\mu]$, $N_{i}$ being the number of reported cases at day i = 1,2,3,4) that we imposed as exposed individuals consecutively before $\frac{1}{R_{2}}+\frac{1}{R_{3}}+\frac{1}{\tau}$ days. For the rationale of such an initial condition, see ^2^.

**Fitting strategy:**

**Step 1:** We generated 1000 parameter sets by sampling the parameters using a uniform distribution around their mean values (Table S3) by 20% in both directions (the fixed parameters are kept fixed). For each of these generated parameters set, we use the case data (total symptomatic cases after data smoothening as explained above and total deaths) from 23^rd^ of February to 16^th^ of March 2020 in a single stretch to calibrate $R_{1}$ by minimizing the cost functions for the data observed until March 16^th^ . The *‘interior-point’* method ^6^ implemented in the *MATLAB* function *‘fmincon’ was used* as an optimization algorithm while carrying out *‘GlobalSearch’* ^7^ with 14 starting points in *MATLAB Release 2018a*. Each of these starting points which represent the initial parameter values of $R_{1}$ from where the search begins for a corresponding optimization for $R_{1}$, is generated randomly from the range of 0 and 1.5 within the *‘GlobalSearch’* platform in *MATLAB Release 2018a*. Among the resulting solutions, only the best solution with the minimal cost function value (normalized sum of squared residuals) was used*.* The, thus, calibrated $R_{1}$ for this span of days (in a single stretch) along with the corresponding parameters in a particular set was used to determine the basic reproduction number $R_{0}$. The logic of such a strategy comes from the fact that the timespan till 16^th^ of March was a period where NPIs were not likely to show an impact.

**Step 2:** Obtain $R_{0}$ with the equation above for each of these parameter sets using the calibrated $R_{1}$.

**Step 3:** Use 100 parameter sets randomly chosen from the 1000 obtained sets (see Step 1) and, for each of these 100 chosen parameter sets, estimate $R_{1}$ (contact frequency dependent transmission rate) in a shifting time-window using a global optimization method and multiple starting points (we use 14 starting points for each performed fitting in each window). For a particular parameter set, while optimising $R_{1}$ for each of the time-windows by sliding the start of the next time-window by one day, we use the same parameter set. For details of the implemented global optimization method, see Step 1.

**Step 4:** Calculate $R_{t}$ (note, $R_{t}$ can be more than $R_{0}$ due to temporal fluctuation) and report for each of the windows and for each of these 100 parameter sets.

**SUPPLEMENTARY FIGURES**

(Material and Methods as in main manuscript)

Population of Ischgl at time-point of study enrollment (n=1867)

including

- seasonal immigrant worker with permanent secondary residence (n=250)
- children (n=340)

## Target population Allocation

## Analysis

Enrolled (n=1534, 82%)

- at study acquisition center (n=1495)
- visited at home (n=41)

## Enrollment

Analysed (n=1473, 79%)

- adults (n=1259, 82%)
- children (n=214, 63%)
- living in 478 households

excluded from analysis: 2 children, who declined participation and 61 participants with incomplete bio-samples

**Supplementary Figure 1. Study enrollment.** The target population for this cross-sectional study consisted of the Ischgl population at the time-point of the survey (n=1867 inhabitants with main residence in Ischgl including 250 seasonal immigrant workers with permanent secondary residence). While 1495 study participants appeared at the study acquisition center, 41 were visited at home by the general practitioner. Due to incomplete bio-samples from 61 individuals and 2 exclusions in the pediatric cohort due to decline of participation, a total of 1473 study participants from 478 households were included into statistical analysis. As the study was anonymous and some individuals did not visit the study center together with the rest of the household members, 106 individuals could not be assigned to a household. Overall participation rate for analysis was 79%.

**Supplementary Figure 2. Correlation of anti-S with anti-N IgG antibodies to SARS-CoV-2.** Anti-S-IgG (Euroimmun) plotted against anti-N-IgG (Abbott) in plasma samples of 1473 study participants with Spearmans correlation coefficient (r) and 95% confidence interval (CI) of the coefficient. Dotted horizontal line represents the cutoff value for Abbott IgG test. Dotted vertical lines indicate negative (<0.8), borderline (0.8-1.1) and positive (>1.1) cutoff values for Euroimmun anti-S-IgG test.

RLU= relative light unit, OD=Optical density

**
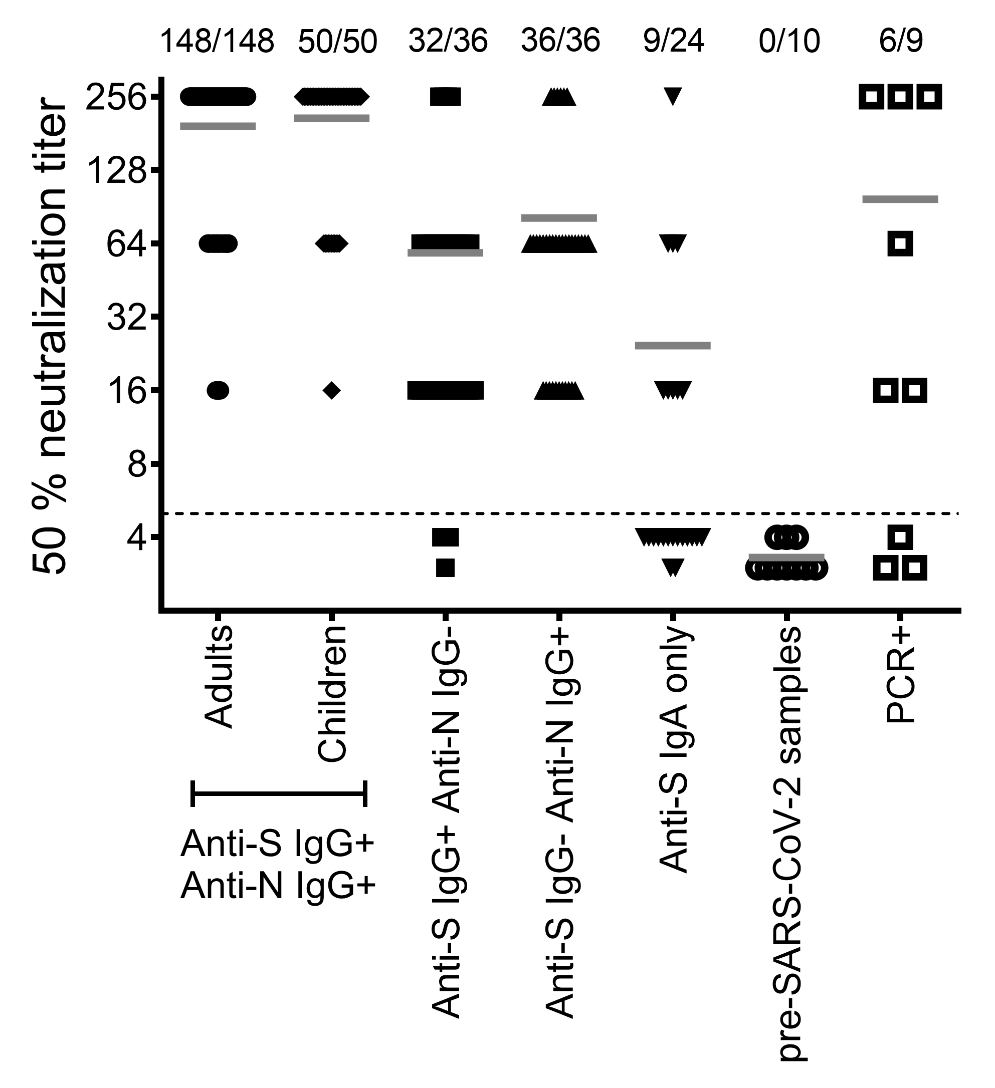
**

**Supplementary Figure 3. Neutralizing antibody titers in seropositive individuals.** 50% neutralization titers were determined using a replication competent SARS-CoV-2 isolate. Numbers above the graph indicate number positive samples/total number tested (n=148 for anti-S IgG+/anti-N IgG+ adults, n=50 for anti-S IgG+/anti-N IgG+ children, n=36 for anti-S IgG+/anti-N IgG-, n=36 for anti-S IgG-/anti-N IgG+, n=24 for only anti-S IgA+, n=10 for plasma samples obtained before the SARS-CoV-2 pandemic, and n=9 for PCR positive individuals found in the study. Among the PCR positive samples were 3 anti-S IgG+/anti-N IgG+, 1 anti-S IgG+/anti-N IgG-, 2 only anti-S IgA+, and 3 anti-S IgG-/anti-N IgG-. Shown are mean and individual samples. Dashed line shows cut-off for the neutralization assay (>1:4).

**
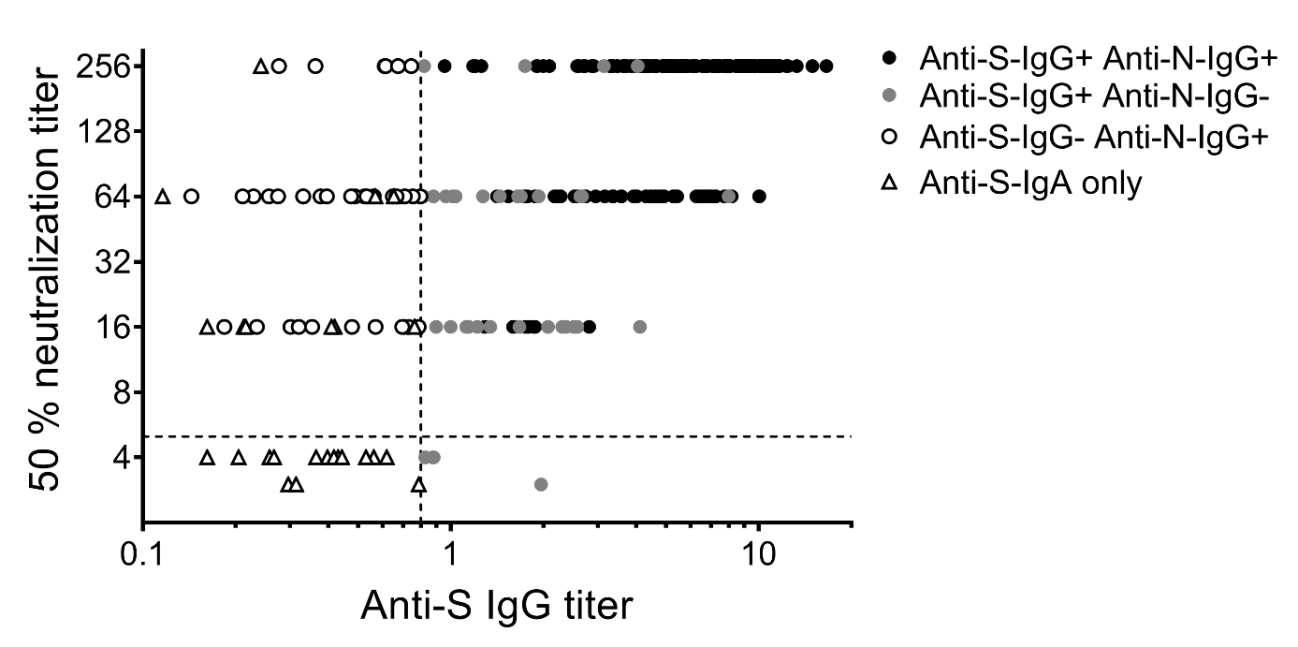
**

**Supplementary Figure 4. Titers of neutralizing antibodies correlate with anti-S IgG levels.** Plasma samples were analyzed via ELISA for titers of Anti-S IgG antibodies and via neutralization assay using replication competent SARS-CoV-2 for 50% neutralization titers. Each data point represents an individual plasma sample; n=201 for anti-S IgG+/anti-N IgG+ (50 children and 151 adults); n= 37 for anti-S IgG+/anti-N IgG-; n=36 for anti-S IgG-/anti-N IgG+; n=26 for solely anti-S IgA positive subjects. Dashed lines show cut-off for neutralization assay (>1:4) and anti-S IgG ELISA (≥0.8).


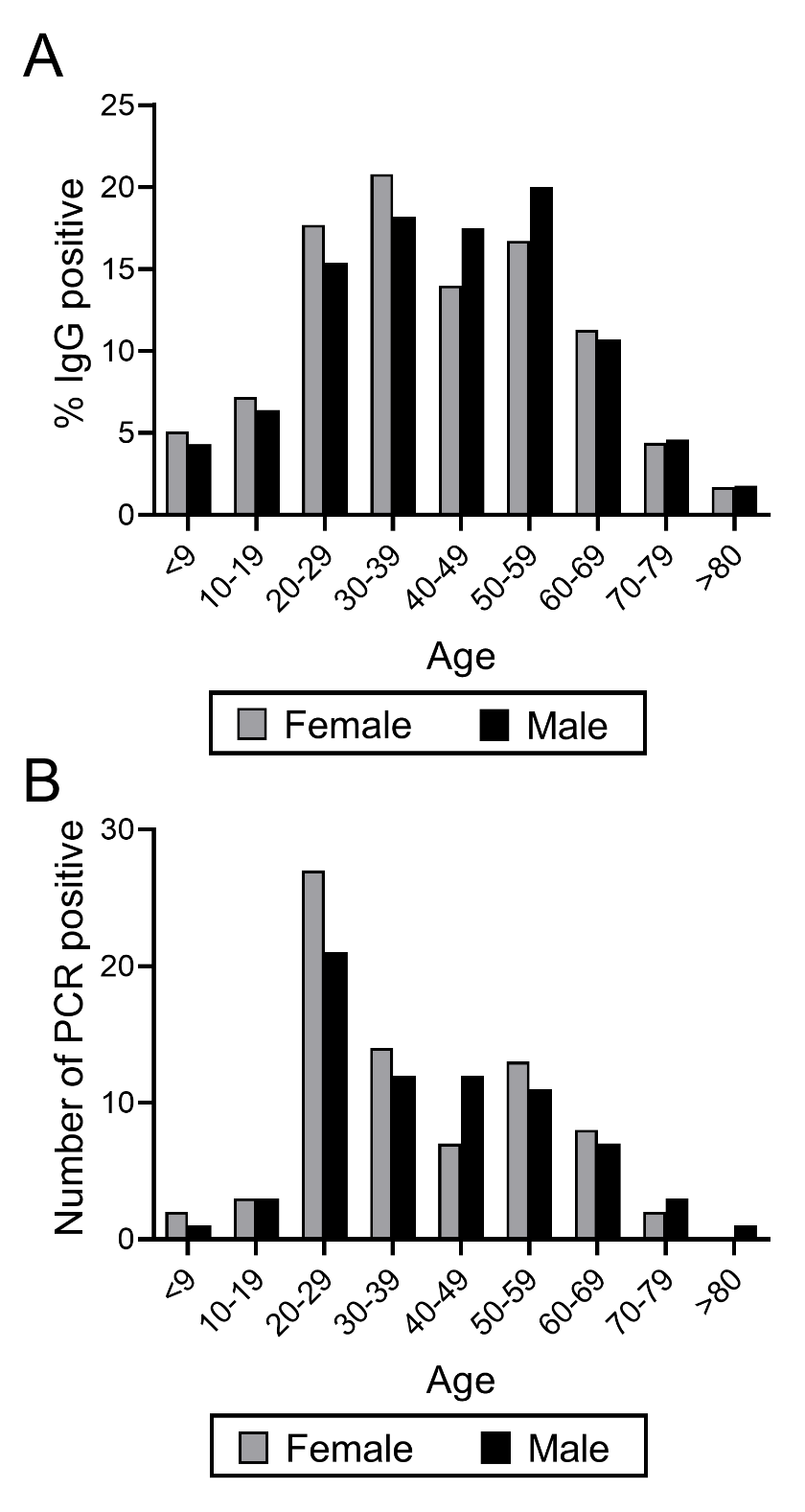


**Supplementary Figure 5. Children likely underdiagnosed by SARS-CoV-2 PCRs performed in Ischgl to a greater extent than adults. a** Age distribution of seroprevalence among study participants in Ischgl, n=1473. y-axis: percent antibody positive of total population, x-axis: age in years. **b** Age-distribution of positive SARS-CoV-2 PCR tests among age- and sex- groups in Ischgl. Data were kindly provided by the Austrian Agency for Health and Food Safety (AGES GmbH). y-axis: percent positive of all SARS-CoV-2 PCR tests performed in Austria, x-axis: age in years.

**
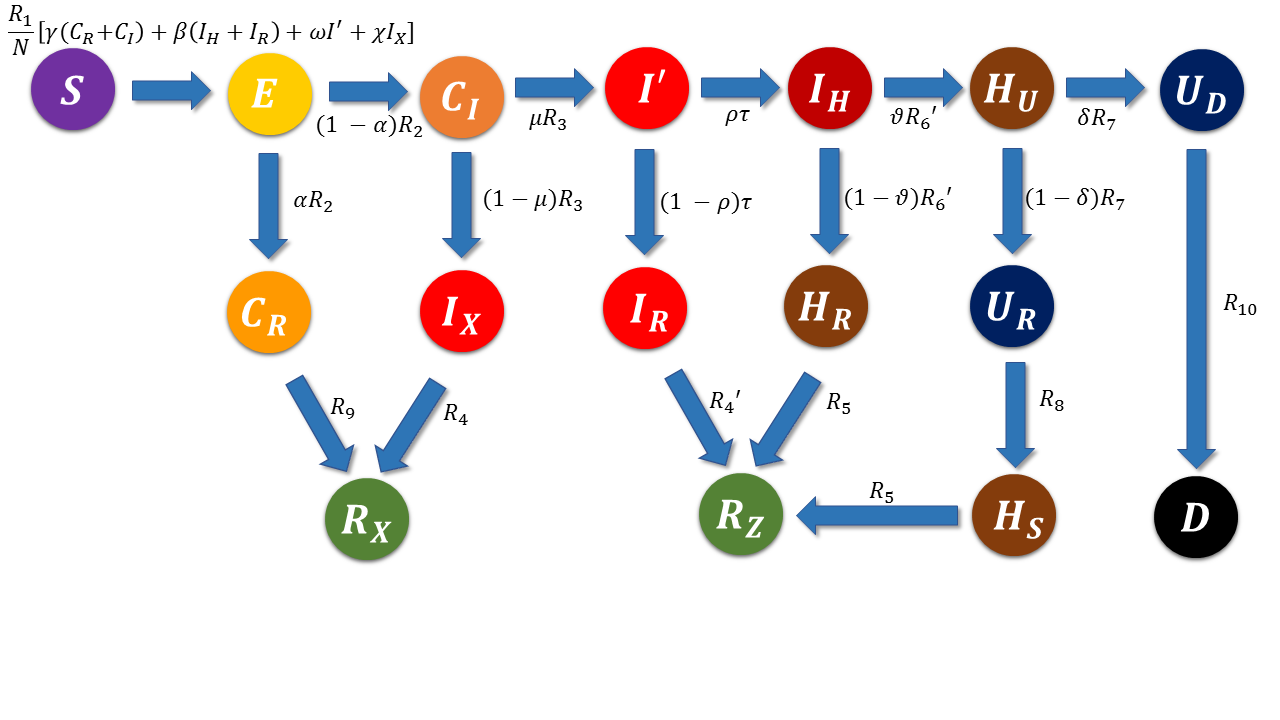
**

**Supplementary Figure 6.** **Model used to study COVID-19 outbreak in Ischgl.** A compartment model ^2^ was calibrated to simulate the dynamics of the outbreak in Ischgl. For information regarding specific compartments, see Supplementary Figure 7.


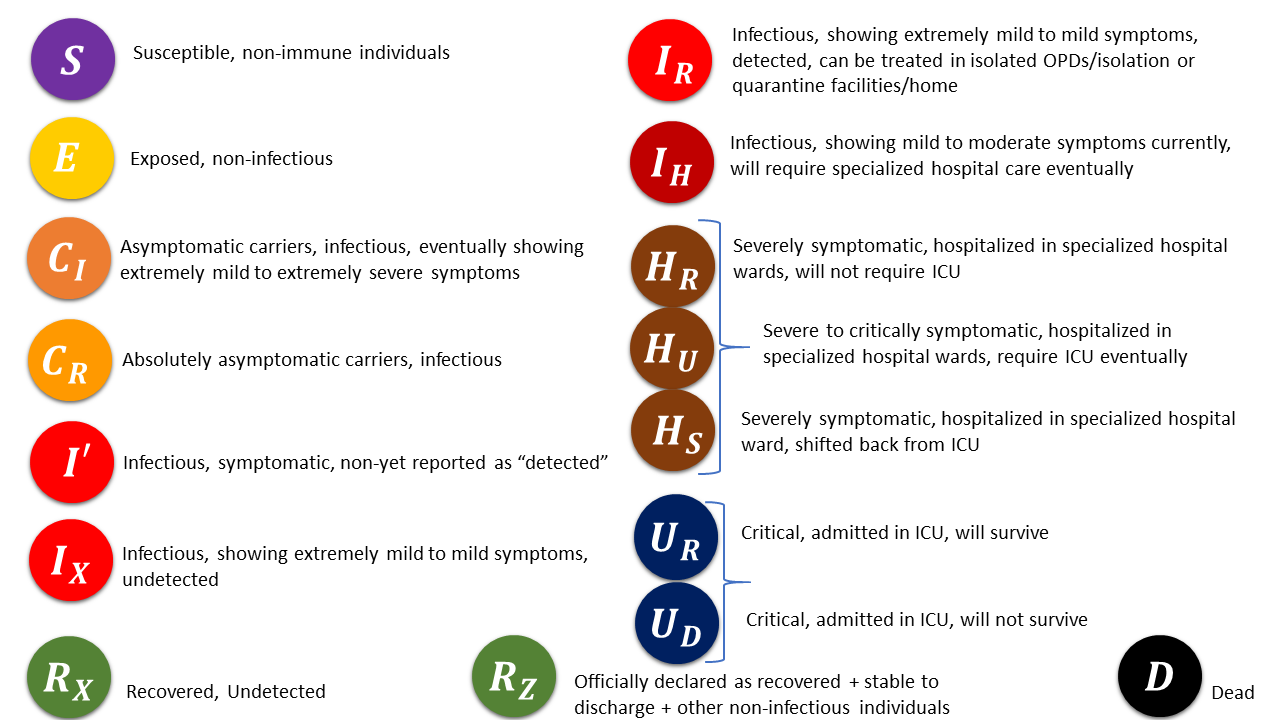


**Supplementary Figure 7.** **Specific description of the compartments** of the model in Supplementary Figure 6.

**
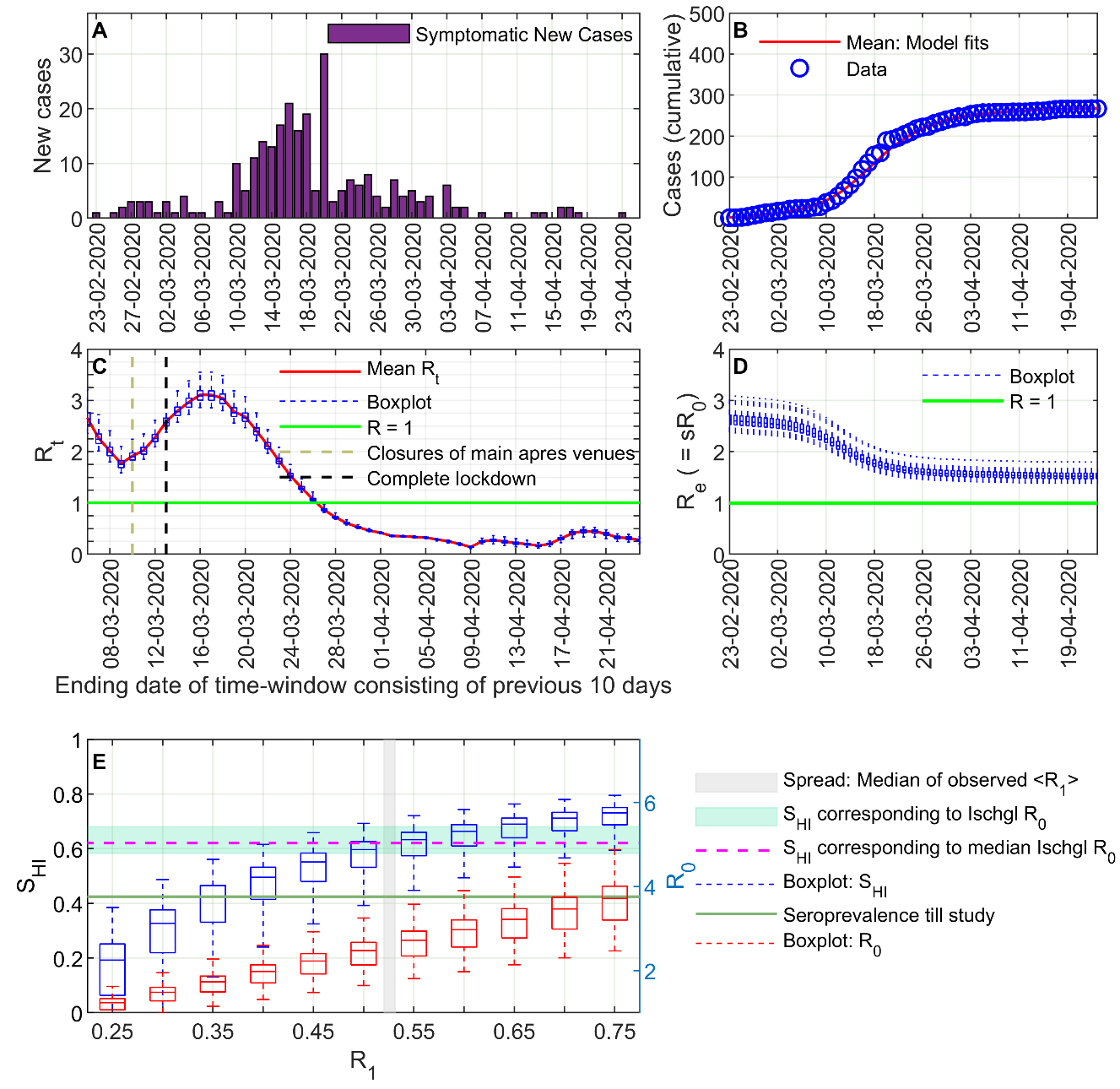
**

**Supplementary Figure 8.** **Analysis based on epidemic curve constructed with temporal data for onset of anosmia/dysgeusia among the seropositive individuals.** The time-course of new COVID-19 cases in Ischgl based on anosmia/dysgeusia among seropositive individuals as obtained from the survey **(a)** was used to determine the daily infection transmission rate ($R_{1}$) for each of the sampled parameter sets (see Supplementary Methods). The calibrated model reproduces the cumulative number of anosmia/dysgeusia cases **(b)** and characterizes the time-dependent reproduction number $R_{t}$ **(c)**. Time-dependent alterations in the effective reproduction number $R_{e}$ **(d)** measured in terms of $R_{e}=sR_{0}$, $s$ being the fraction of the susceptible population at a certain time. **(e)** Seroprevalence necessary for herd immunity ($S_{HI}$, green shaded region) and basic reproduction number ($R_{0}$, red box plots) are shown as functions of $R_{1}$. The analysis was done with 100 parameter sets following calibration of $R_{1}$ to best describe the case numbers until March 16^th^ at a single complete stretch (see fitting strategy in the Supplementary Methods). $R_{0}$ in Ischgl was between 2.4 to 3.1, with a median value of 2.6, which corresponds to a median value of 61.5% seroprevalence to reach herd immunity ($S_{HI}$ corresponding to median $R_{0}$, purple line). This is consistent with herd-immunity seroprevalence (blue box plot) derived from the medians of the mean contact dependent daily transmission rate $<R_{1}>$ for the time-windows spanning February 23^rd^ – March 16^th^ and February 28^th^ – March 16^th^ (grey patch, bounded by these medians). The observed seroprevalence (green line) as of April 2020 is lower than the thresholds. We assume that the seropositive individuals who reported their onset of symptoms before February 23^rd^, 2020 during the survey were not symptomatic due to COVID-19 at that time, but rather had an asymptomatic course (in terms of anosmia/dysgeusia in this case) of COVID-19 later. In addition, for this analysis, cases that did not report onset of anosmia/dysgeusia from February 23^rd^, 2020 were also considered to be asymptomatic due to significantly higher OR for anosmia/dysgeusia as compared to any other symptom. In boxplots median, interquartile range and whiskers with maximal 1.5× interquartile range are shown.


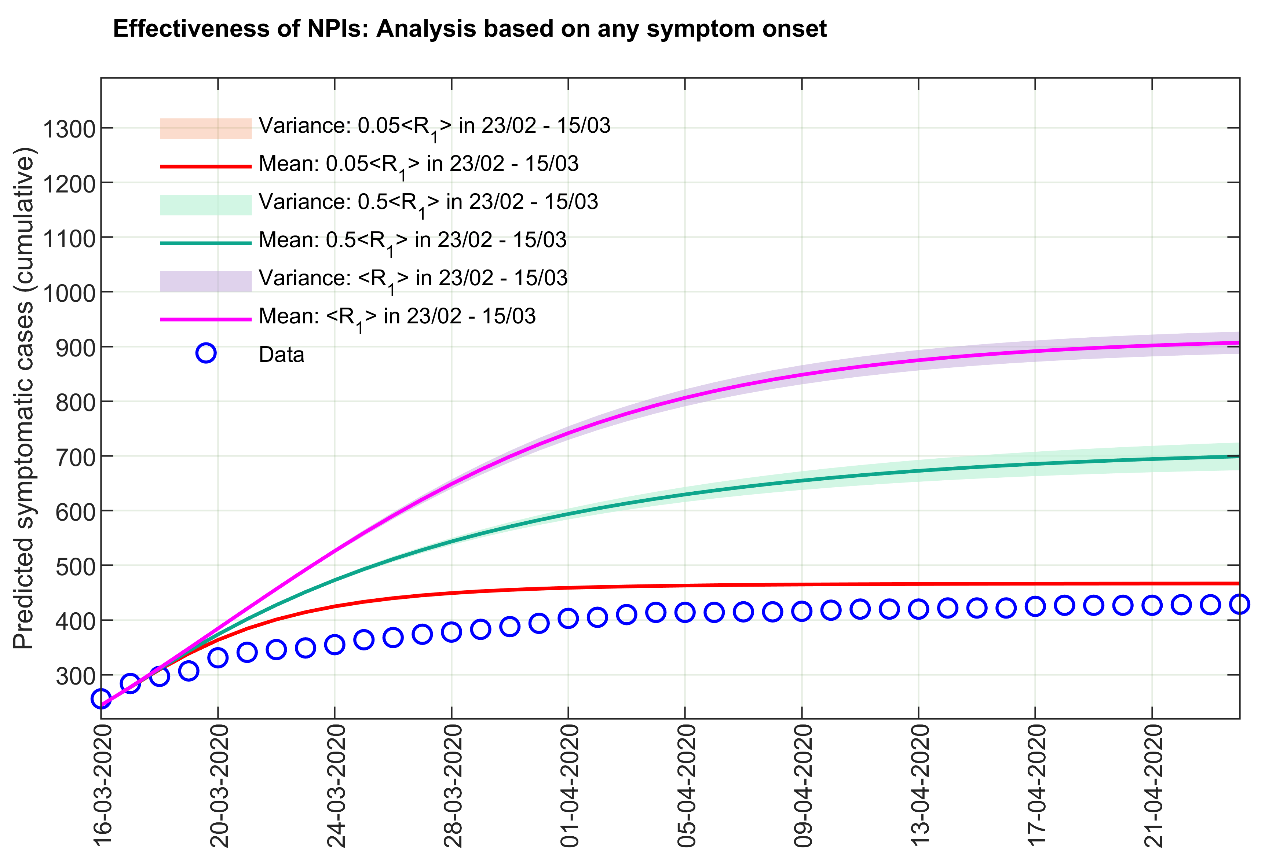


**Supplementary Figure 9. Mathematical modelling suggests the effectiveness of the implemented NPIs in Ischgl.** Different scenarios for the future time-course of the outbreak after mid-March were projected until April 24^th^, 2020 using three different contact dependent transmission rates ($R_{1}$), viz., 100% (pink), 50% (green) and 5% (red) of the observed $<R_{1}>$ during the time spanning February 23^rd^ to March 15^th^, 2020, along with the survey-oriented Ischgl case data (blue dots) during the same period. The lines depict the average time course and the shaded areas show the variance of the simulated results. A substantial difference between the observed data and simulated results with unrestricted transmission rate of $<R_{1}>$ during February 23^rd^ to March 15^th^, 2020 suggested the effectiveness of NPIs in Ischgl.

**
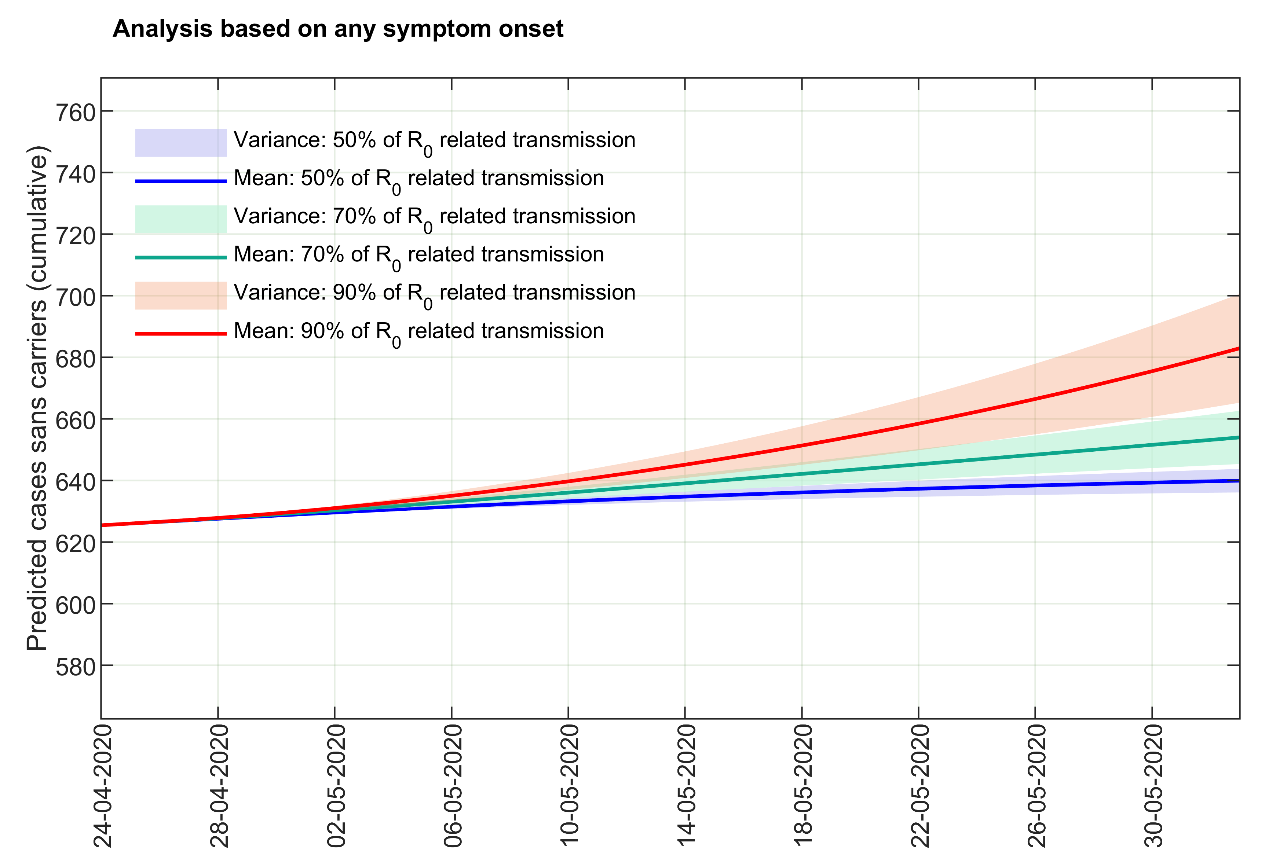
**

**Supplementary Figure 10. Without NPIs, if the ski resort continued its activity as the way it was doing before closures, the village could face a new outbreak even after April 2020.** Different scenarios for the future time-course of the outbreak after April 24^th^, 2020 were projected until start of June 2020 using three different reproduction numbers calculated using 90% (red), 70% (green) and 50% (blue) of the calibrated $R_{1}$used to calculate the basic reproduction number $R_{0}$as obtained from fitting the Ischgl case data till March 16^th^ when the virus was spreading without the effect of the restrictions in place (see fitting strategy in the supplementary Methods). The lines depict the average time course, and the shaded areas show the variance of the simulated results. A substantial increase in the predicted number of cases in case of viral spreading with a value close to $R_{0}$suggested that the ski resort could face a new outbreak without the NPIs in place. This also supported that the village was still away from achieving herd immunity.

**
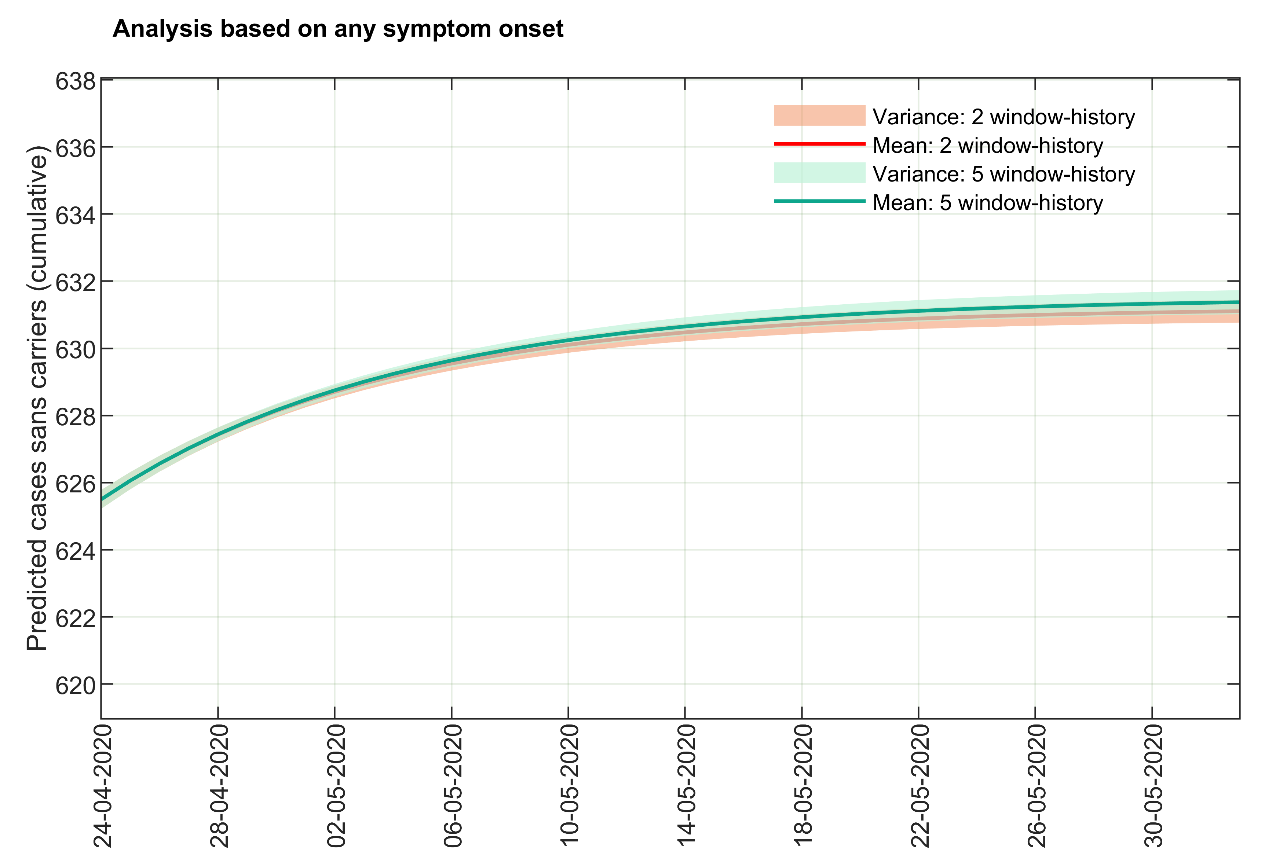
**

**Supplementary Figure 11.** **Keeping the transmission rate as it is in the ski resort after April 2020 would ensure no significant increase in new infections.** Different scenarios for the future time-course of the outbreak after April 24^th^, 2020 were projected until start of June 2020 using the history of contact dependent transmission rates ($R_{1}$) in two recent past time-windows, viz., $<R_{1}>$ in the last 5 (green) and the last 2 (red) time-windows ending on April 24^th^,2020. The lines depict the average time course, and the shaded areas show the variance of the simulated results. Both scenarios did not result in a substantial increase in the predicted number of cases in the ski resort.

**
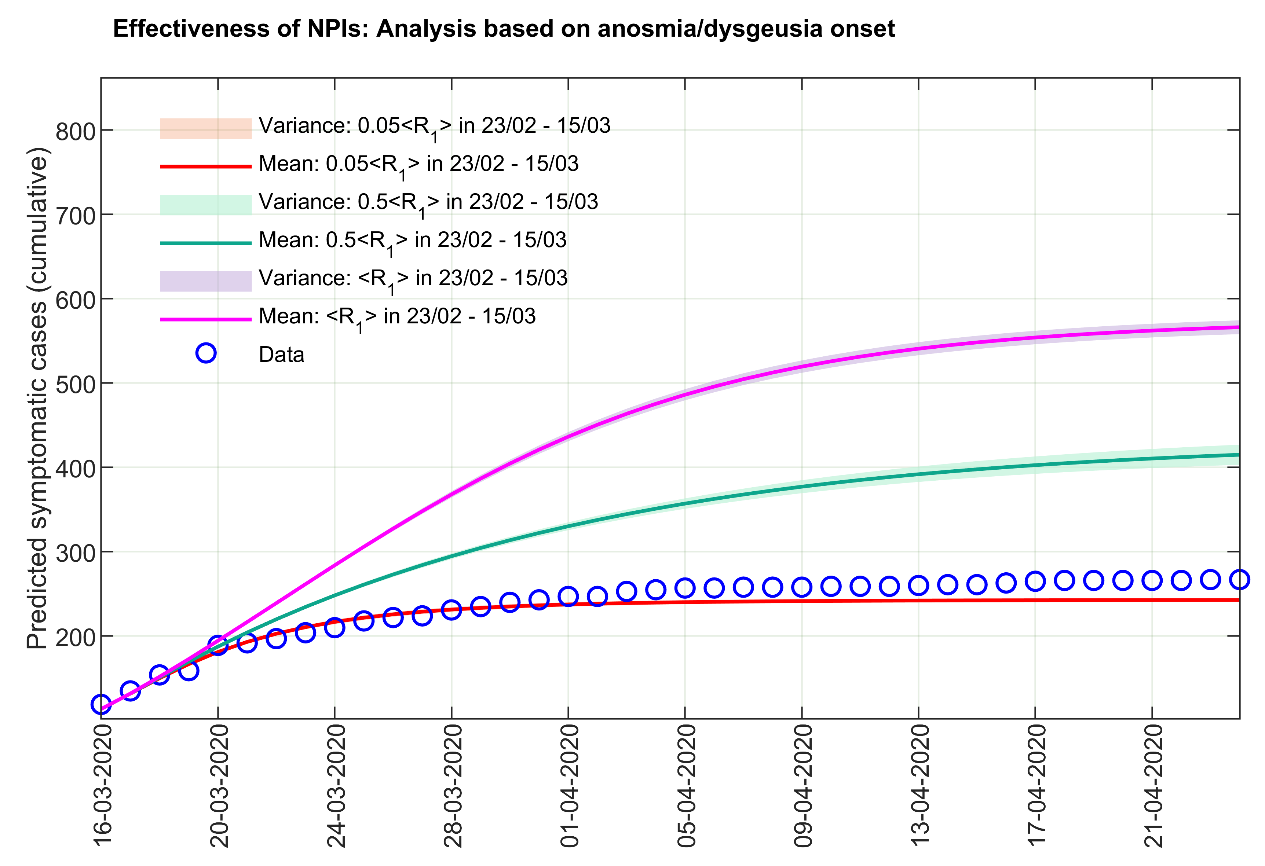
**

**Supplementary Figure 12. Mathematical modelling suggests the effectiveness of the implemented NPIs in Ischgl using anosmia/dysgeusia data.** Different scenarios for the future time-course of the outbreak after mid-March were projected until April 24^th^, 2020 using three different contact dependent transmission rates ($R_{1}$), viz., 100% (pink), 50% (green) and 5% (red) of the observed $<R_{1}>$ during the time spanning February 23^rd^ to March 15^th^, 2020, along with the survey-oriented Ischgl anosmia/dysgeusia case data (blue dots) during the same period. The lines depict the average time course, and the shaded areas show the variance of the simulated results. A substantial difference between the observed anosmia/dysgeusia data and simulated results with unrestricted transmission rate of $<R_{1}>$ during February 23^rd^ to March 15^th^, 2020 suggested the effectiveness of NPIs in Ischgl.

**
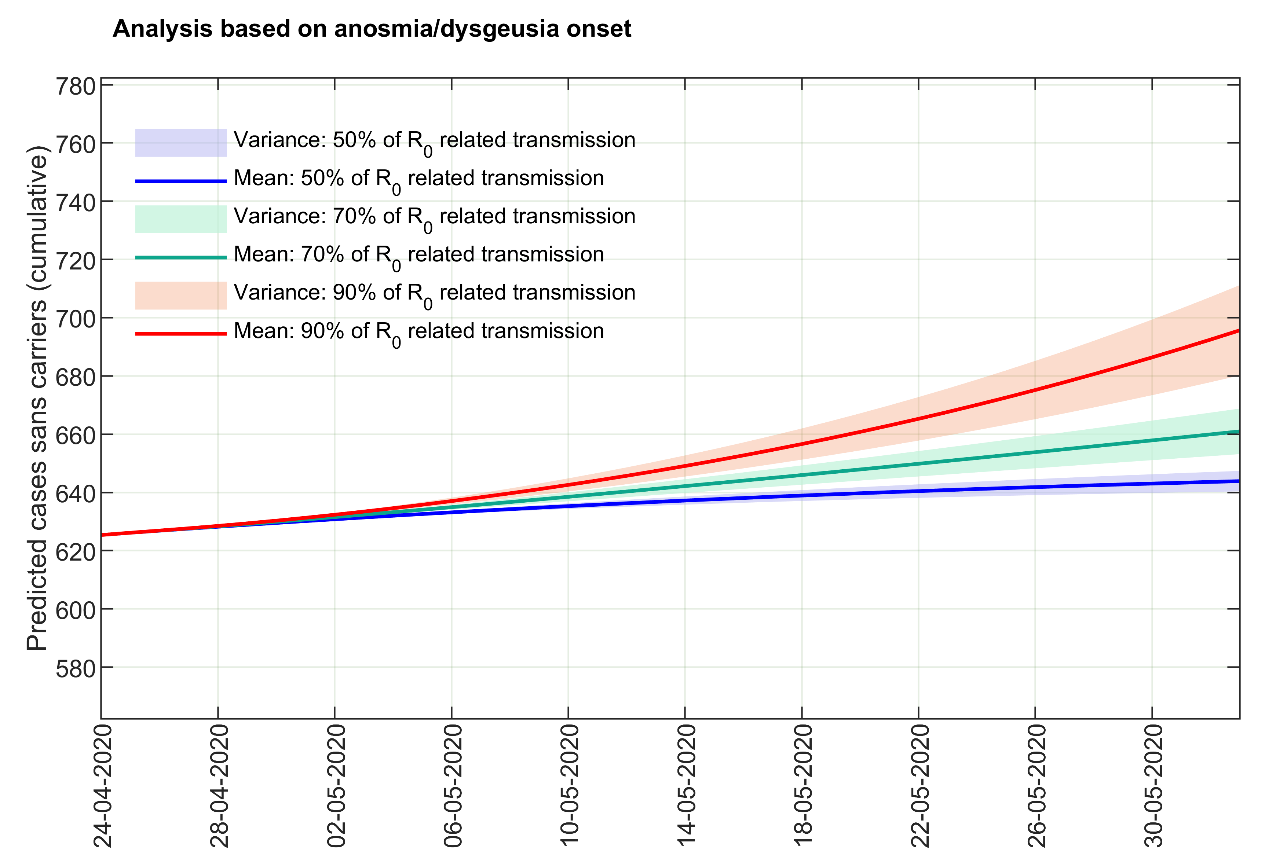
**

**Supplementary Figure 13. Without NPIs, if the ski resort continued its activity as the way it was doing before closures, the village could face a new outbreak even after April 2020.** Different scenarios for the future time-course of the outbreak after April 24^th^, 2020 were projected until start of June 2020 using three different reproduction numbers calculated using 90% (red), 70% (green) and 50% (blue) of the calibrated $R_{1}$ used to calculate the basic reproduction number $R_{0}$as obtained from fitting the Ischgl anosmia/dysgeusia case data till mid-March when the virus was spreading without restrictions. The lines depict the average time course, and the shaded areas show the variance of the simulated results. A substantial increase in the predicted number of cases in case of viral spreading with a value close to $R_{0}$suggested that the ski resort could face a new outbreak without the NPIs in place. This also supported that the village was still away from achieving herd immunity.

**
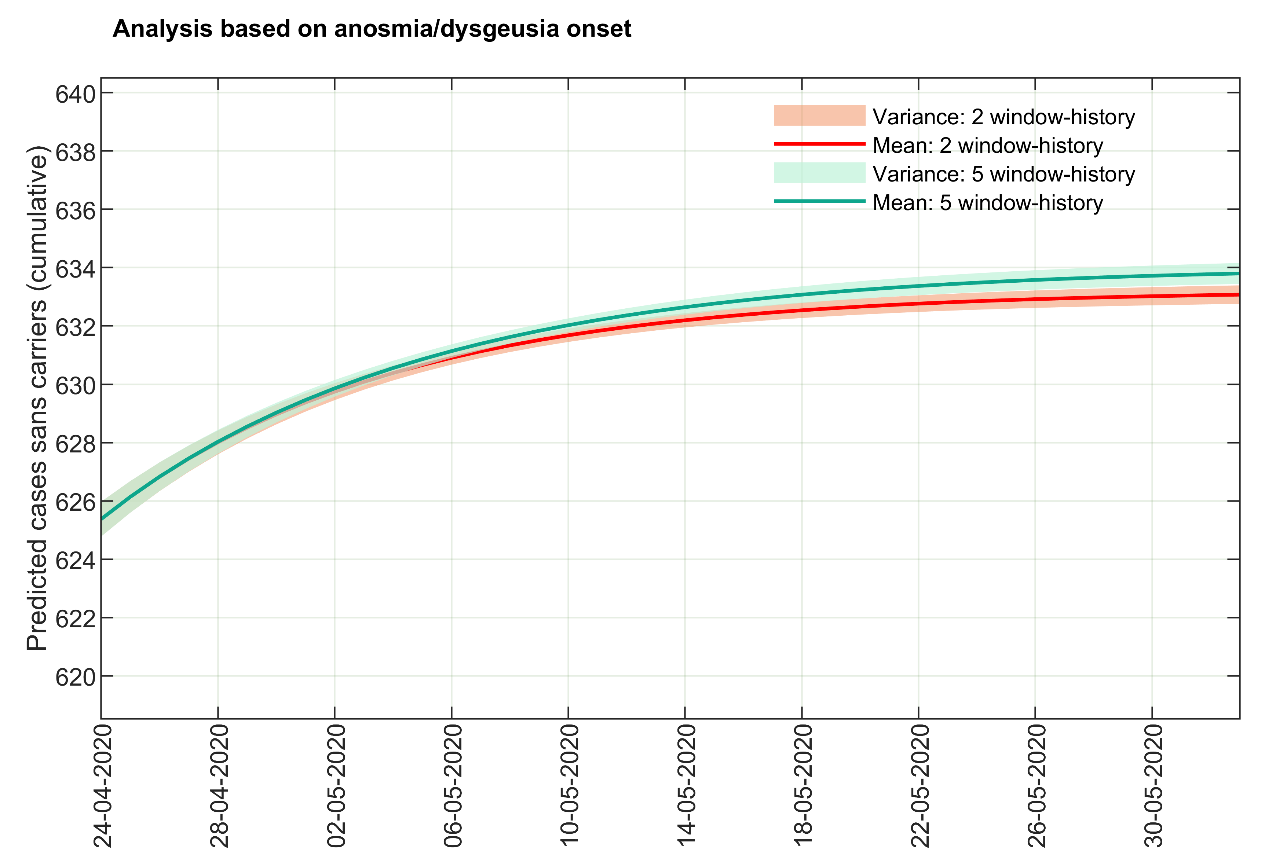
**

**Supplementary Figure 14. Keeping the transmission rate as it is in the ski resort after April 2020 would ensure no significant increase in new infections.** Different scenarios for the future time-course of the outbreak after April 24^th^, 2020 were projected until start of June 2020 using the history of contact dependent transmission rates ($R_{1}$) in two recent past time-windows, viz., $<R_{1}>$ in the last 5 (green) and the last 2 (red) time-windows ending on April 24^th^ 2020. The lines depict the average time course, and the shaded areas show the variance of the simulated results. Both scenarios did not result in a substantial increase in the predicted number of cases in the ski resort.


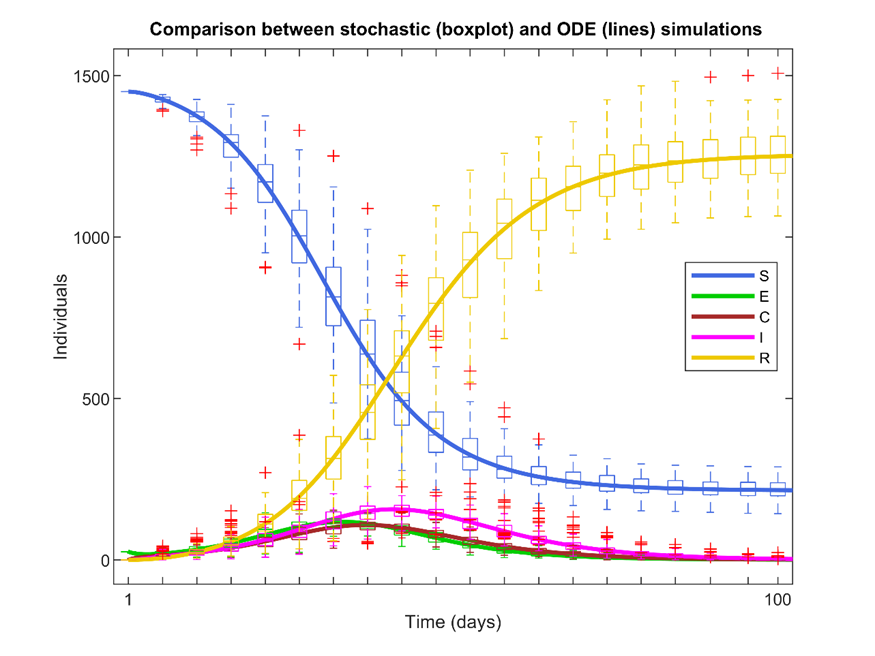


**Supplementary Figure 15**. **Effect of stochasticity.** The effect of stochasticity was tested for such a small population for the same model using the Gillespie algorithm ^8^. We find our ODE simulations to capture well the median behavior of the stochastic system, thereby validating our approach. In the figure, C, I, R represent the sum of all compartments of the carrier, infectious and recovered population, respectively (see Supplementary Figure 6). As shown, the ODE solutions match almost exactly the trend of the median calculated out of the stochastic simulations (100 simulations were carried and the results are shown in the box plots). Boxplots show median, interquartile range and whiskers with maximal 1.5× interquartile range.

**SUPPLEMENTARY TABLES**

**Supplementary Table 1. Characterizing study participants with current PCR-confirmed SARS-CoV-2 infection (n=9)**

| **Age** | **PCR CT** | **Antibody** | | | | **Previous symptoms** | **Time since symptom onset** | **Current symptoms** |
| --- | --- | --- | --- | --- | --- | --- | --- | --- |
|  |  | Anti S IgA (ODratio*) | Anti S IgG (ODratio)* | Anti N IgG  (RLU)** | Neutralization assay ^§^ |  |  |  |
| 18-24 | 38.1 | 0.5 | 0.22 | 0.1 | ≤1:4 | None | - | None |
| 35-44 | 38.9 | 4.21 | 0.79 | 0.1 | ≤1:4 | None | - | None |
| 55-64 | 36.1 | 1.29 | 5.72 | 7 | 1:256 | Anosmia, GI symptoms | 39 days | None |
| 55-64 | 37.2 | 2.37 | 7.44 | 6.3 | 1:256 | None | - | None |
| 14-17 | 38.2 | 0.11 | 0.11 | 0 | 1:16 | None | - | None |
| 6-9 | 35.2 | 1.33 | 0.41 | 0 | 1:16 | None | - | None |
| 10-13 | 38.2 | 0.62 | 0.29 | 0 | ≤1:4 | GI symptoms | 37 days | None |
| >75 | 33.3 | 4.73 | 8 | 9.5 | 1:256 | Cough, fever, breathing difficulties, sore throat, anosmia, dysgeusia, GI symptoms | 25 days | None |
| 25-34 | 30.3 | 0.72 | 0.96 | 0.3 | 1:64 | Anosmia, dysgeusia | 35 days | None |

# PCR positivity known (32days before study begin); * ODratio <0.8 =negative. 0.8 - 1.1=borderline. >1.1= positive ; ** RLU>1.40 positive; ^§^cutoff: >1:4=positive, ≤1:4=negative
CT=cycle threshold. OD= optical density. RLU= relative light unit. S= spike protein. N= nucleocapsid protein

| **Sero status of Household** | **Number of Households** | **% within Household Type** | **% of All Household** |
| --- | --- | --- | --- |
| **Household with no children** | | |  |
| **Mixed** | 108 | 30.5 | 22.6 |
| **Negative** | 150 | 42.4 | 31.4 |
| **Positive** | 96 | 27.1 | 20.1 |
| **Household with one child** | | |  |
| **Mixed** | 30 | 60 | 6.3 |
| **Negative** | 15 | 30 | 3.1 |
| **Positive** | 5 | 10 | 1.0 |
| **Household with 2+ children** | | |  |
| **Mixed** | 49 | 66.2 | 10.3 |
| **Negative** | 19 | 25.7 | 4.0 |
| **Positive** | 6 | 8.1 | 1.3 |
| **Total** | 478 |  | 100 |

**Supplementary Table 2 Household level analysis of sero status by number of children in household**

**Supplementary Table 3.** Estimated means and their bounds used to determine the parameter sets, each of which is then used to calibrate $R_{1}$ within the bounds of 0 and 1.5. See fitting strategy in the Supplementary Methods. For details of parameter estimation from literature, see ^2^.

| **Parameter** | **Parameter set from literature** | |
| --- | --- | --- |
|  | Mean | Variation |
| $\boldsymbol{R}_{\mathbf{2}}$ ^9-11^ | $\frac{1}{3.2}$ | 20% around mean |
| $\boldsymbol{R}_{\boldsymbol{3}}$ ^9^ | Determined by: $\frac{1}{R_{3}}=5.2- \frac{1}{R_{2}}$  Pre-symptomatic infectious period is given by the subtraction of the latent period from the incubation period. | |
| $\boldsymbol{R}_{\mathbf{4}}$R_4 ^12-14^ | $\frac{1}{7}$ | 20% around mean |
| $\boldsymbol{R}_{\mathbf{4}}\boldsymbol{'}$ | Determined by: $\frac{1}{R_{4}'}=\frac{1}{R_{4}}-\frac{1}{\tau}$  Time to recovery from acknowledged (detected) case is given by the subtraction of the time to recognition of symptoms (1/τ) from the time to recovery from symptom onset | |
| $\boldsymbol{R}_{\mathbf{5}}$R_5 ^15,16^ | $\frac{1}{8}$ | 20% around mean |
| $\boldsymbol{R}_{\mathbf{6}}$R_6 ^17^ | $\frac{1}{4.25}$ | 20% around mean |
| $\boldsymbol{R}_{\mathbf{6}}\boldsymbol{'}$ | Determined by: $\frac{1}{R_{6}'}=\frac{1}{R_{6}}-\frac{1}{\tau}$  Time to hospitalization from acknowledged (detected) case is given by the subtraction of the time to recognition of symptoms (1/τ) from the time to hospitalization from symptom onset | |
| $\boldsymbol{R}_{\mathbf{7}}$R_7 ^16,17^ | $\frac{1}{4.25}$ | 20% around mean |
| $\boldsymbol{R}_{\mathbf{8}}$R_8 ^15^ | $\frac{1}{9}$ | 20% around mean |
| $\boldsymbol{R}_{\boldsymbol{9}}$ | Determined by: $\frac{1}{R_{9}}=\frac{1}{R_{3}}+(0.5 \times\frac{1}{R_{4}})$  This relation results from the assumption that the asymptomatic infectious people becomes non-infectious randomly at some point during a similar duration of symptomatic recovery period. See ^2^. | |
| $\boldsymbol{R}_{\mathbf{10}}$R_10 ^17,18^ | $\frac{1}{7.43}$ | 20% around mean |
| $\boldsymbol{\beta}$ β | Assumed: sampled from the range 0.05 – 0.5 | |

**Supplementary Table 4. Robustness analysis.** Estimated bounds of $R_{0}$ with its median value and seroprevalence corresponding to the median $R_{0}$ values for different case onset data and different number of parameter-sets.

| **Data** | **Number of Parameter Sets** | **Minimum** $\boldsymbol{R}_{\boldsymbol{0}}$ | **Maximum** $\boldsymbol{R}_{\boldsymbol{0}}$ | **Median** $\boldsymbol{R}_{\boldsymbol{0}}$ | **Median Seroprevalence for Herd Immunity** |
| --- | --- | --- | --- | --- | --- |
| **Onset of any symptom since February 23^rd^, 2020** | 100 | 2.2 | 3.1 | 2.5 | 60% |
|  | 500 | 2.2 | 3.1 | 2.5 | 60% |
|  | 1000 | 2.2 | 3.1 | 2.6 | 61.5% |
| **Onset of anosmia/**  **Dysgeusia**  **since February 23^rd^, 2020** | 100 | 2.4 | 3.1 | 2.6 | 61.5% |
|  | 500 | 2.4 | 3.1 | 2.7 | 63% |
|  | 1000 | 2.4 | 3.1 | 2.6 | 61.5% |

**SUPPLEMENTARY REFERENCES:**

1 Hoffmann, M. *et al.* SARS-CoV-2 Cell Entry Depends on ACE2 and TMPRSS2 and Is Blocked by a Clinically Proven Protease Inhibitor. *Cell* **181**, 271-280 e278, doi:10.1016/j.cell.2020.02.052 (2020).

2 Khailaie, S. *et al.* Development of the reproduction number from coronavirus SARS-CoV-2 case data in Germany and implications for political measures. *BMC Med* **19**, 32, doi:10.1186/s12916-020-01884-4 (2021).

3 Diekmann, O., Heesterbeek, J. A. & Metz, J. A. On the definition and the computation of the basic reproduction ratio R0 in models for infectious diseases in heterogeneous populations. *J Math Biol* **28**, 365-382, doi:10.1007/BF00178324 (1990).

4 van den Driessche, P. & Watmough, J. Reproduction numbers and sub-threshold endemic equilibria for compartmental models of disease transmission. *Math Biosci* **180**, 29-48, doi:10.1016/s0025-5564(02)00108-6 (2002).

5 van den Driessche, P. & Watmough, J. in *Mathematical Epidemiology. Lecture Notes in Mathematics* Vol. 1945 (Springer, Berlin, Heidelberg, 2008).

6 Byrd, R. H., Hribar, M. E. & Nocedal, J. An Interior Point Algorithm for Large-Scale Nonlinear Programming. *SIAM J. Optim.* **9**, 877-900, doi:<https://doi.org/10.1137/S1052623497325107> (1999).

7 Ugray, Z. *et al.* Scatter Search and Local NLP Solvers: A Multistart Framework for Global Optimization. *INFORMS Journal on Computing* **19**, doi:<https://doi.org/10.1287/ijoc.1060.0175> (2007).

8 Gillespie, D. T. Exact stochastic simulation of coupled chemical reactions. *The Journal of Physical Chemistry* **81**, 2340-2361, doi:10.1021/j100540a008 (1977).

9 Li, Q. *et al.* Early Transmission Dynamics in Wuhan, China, of Novel Coronavirus-Infected Pneumonia. *N Engl J Med* **382**, 1199-1207, doi:10.1056/NEJMoa2001316 (2020).

10 He, X. *et al.* Temporal dynamics in viral shedding and transmissibility of COVID-19. *Nat Med* **26**, 672-675, doi:10.1038/s41591-020-0869-5 (2020).

11 Hao, X. *et al.* Reconstruction of the full transmission dynamics of COVID-19 in Wuhan. *Nature* **584**, 420-424, doi:10.1038/s41586-020-2554-8 (2020).

12 Wolfel, R. *et al.* Virological assessment of hospitalized patients with COVID-2019. *Nature* **581**, 465-469, doi:10.1038/s41586-020-2196-x (2020).

13 Clifford, S. *et al.* Strategies to reduce the risk of SARS-CoV-2 re-introduction from international travellers. *medRxiv*, 2020.2007.2024.20161281, doi:10.1101/2020.07.24.20161281 (2020).

14 Bullard, J. *et al.* Predicting Infectious Severe Acute Respiratory Syndrome Coronavirus 2 From Diagnostic Samples. *Clin Infect Dis* **71**, 2663-2666, doi:10.1093/cid/ciaa638 (2020).

15 *Epidemiologischer Steckbrief zu SARS-CoV-2 und COVID-19*, <<https://www.rki.de/DE/Content/InfAZ/N/Neuartiges_Coronavirus/Steckbrief.html>> (Stand: 19.04.2021).

16 *International Severe Acute Respiratory and Emerging Infections Consortium COVID-19 Report: 13 July 2020*, <<https://media.tghn.org/medialibrary/2020/07/ISARIC_Data_Platform_COVID-19_Report_13JUL20.pdf>.> (

17 Dreher, M. *et al.* The Characteristics of 50 Hospitalized COVID-19 Patients With and Without ARDS. *Dtsch Arztebl Int* **117**, 271-278, doi:10.3238/arztebl.2020.0271 (2020).

18 Khalili, M. *et al.* Epidemiological Characteristics of COVID-19; a Systemic Review and Meta-Analysis. *medRxiv*, 2020.2004.2001.20050138, doi:10.1101/2020.04.01.20050138 (2020).
