# Supplementary material for: High SARS-CoV-2 seroprevalence in children and adults in the Austrian ski resort of Ischgl
Source: Commun Med (Lond). 2021 Jun 30;1:4. doi: 10.1038/s43856-021-00007-1 (PMC8633917; doi:10.1038/s43856-021-00007-1)
Supplement: Supplementary file 2 — Description of Additional Supplementary Files [file 43856_2021_7_MOESM2_ESM.pdf]

Description of Additional Supplementary Files

Supplementary Data 1

Source data underlying the graphs and charts presented in Figure 1

Source data underlying the graphs and charts presented in Figures 2 and 3 available at https://doi.org/10.5281/zenodo.4704076
