# Supplementary material for: High SARS-CoV-2 seroprevalence in children and adults in the Austrian ski resort of Ischgl
Source: Commun Med (Lond). 2021 Jun 30;1:4. doi: 10.1038/s43856-021-00007-1 (PMC8633917; doi:10.1038/s43856-021-00007-1)
Supplement: Supplementary file 4 — Reporting Summary [file 43856_2021_7_MOESM4_ESM.pdf]

nature research

Corresponding author(s): Michael Meyer-Hermann
Dorothee von Laer

Last updated by author(s): May 4, 2021

Reporting Summary

Nature Research wishes to improve the reproducibility of the work that we publish. This form provides structure for consistency and transparency in reporting. For further information on Nature Research policies, see our Editorial Policies and the Editorial Policy Checklist.

Statistics

For all statistical analyses, confirm that the following items are present in the figure legend, table legend, main text, or Methods section.

n/a	Confirmed	
☐	☒	The exact sample size (*n*) for each experimental group/condition, given as a discrete number and unit of measurement
☐	☒	A statement on whether measurements were taken from distinct samples or whether the same sample was measured repeatedly
☐	☒	The statistical test(s) used AND whether they are one- or two-sided<br>*Only common tests should be described solely by name; describe more complex techniques in the Methods section.*
☐	☒	A description of all covariates tested
☒	☐	A description of any assumptions or corrections, such as tests of normality and adjustment for multiple comparisons
☐	☒	A full description of the statistical parameters including central tendency (e.g. means) or other basic estimates (e.g. regression coefficient) AND variation (e.g. standard deviation) or associated estimates of uncertainty (e.g. confidence intervals)
☐	☒	For null hypothesis testing, the test statistic (e.g. *F*, *t*, *r*) with confidence intervals, effect sizes, degrees of freedom and *P* value noted<br>*Give P values as exact values whenever suitable.*
☒	☐	For Bayesian analysis, information on the choice of priors and Markov chain Monte Carlo settings
☒	☐	For hierarchical and complex designs, identification of the appropriate level for tests and full reporting of outcomes
☐	☒	Estimates of effect sizes (e.g. Cohen's *d*, Pearson's *r*), indicating how they were calculated

Our web collection on statistics for biologists contains articles on many of the points above.

Software and code

Policy information about availability of computer code

Data collection	SPSS version 26 (Chicago, Illinois, USA), PGS-IT Systems
Data analysis	Data were analysed using Stata 15/MP, StataCorp LLC, SPSS version 26 (Chicago, Illinois, USA), GraphPad Prism version 8.2.0, The simulations and subsequent analysis for the epidemiological model have been performed in MATLAB Release 2018a. A detailed description of our algorithm, analysis and used inbuilt functions if any have been provided in the supplementary appendix for transparency and reproducibility of our study.

For manuscripts utilizing custom algorithms or software that are central to the research but not yet described in published literature, software must be made available to editors and reviewers. We strongly encourage code deposition in a community repository (e.g. GitHub). See the Nature Research guidelines for submitting code & software for further information.

Data

Policy information about availability of data

All manuscripts must include a data availability statement. This statement should provide the following information, where applicable:

- Accession codes, unique identifiers, or web links for publicly available datasets
- A list of figures that have associated raw data
- A description of any restrictions on data availability

All simulation results and source code for the implementation of the model and related algorithms are freely available at https://doi.org/10.5281/zenodo.4704076. Source data for Figure 1 is available as Supplementary Data and for Figure 2 and 3 at https://doi.org/10.5281/zenodo.4704076 17. Upon publication of our study, all other data will be available from the corresponding authors upon receipt of a suitable request.

Field-specific reporting

Please select the one below that is the best fit for your research. If you are not sure, read the appropriate sections before making your selection.

☒ Life sciences ☐ Behavioural & social sciences ☐ Ecological, evolutionary & environmental sciences

For a reference copy of the document with all sections, see nature.com/documents/nr-reporting-summary-flat.pdf

Life sciences study design

All studies must disclose on these points even when the disclosure is negative.

Sample size	Population invited to study n=1867, enrolled n=1534, analysed n=1473
Data exclusions	n=61 excluded from analysis due to incomplete samples set, n=106 individuals were removed from the household analysis due to missing household information.
Replication	not relevant to study as cross-sectional study, for the modelling extensive testing of robustness has been performed as described in the supplement
Randomization	not relevant to study as the whole population of Ischgl was invited to this cross-sectional study
Blinding	not relevant to study as cross-sectional study

Reporting for specific materials, systems and methods

We require information from authors about some types of materials, experimental systems and methods used in many studies. Here, indicate whether each material, system or method listed is relevant to your study. If you are not sure if a list item applies to your research, read the appropriate section before selecting a response.

Materials & experimental systems

n/a	Involved in the study	
☒	☐	Antibodies
☐	☒	Eukaryotic cell lines
☒	☐	Palaeontology and archaeology
☒	☐	Animals and other organisms
☐	☒	Human research participants
☒	☐	Clinical data
☒	☐	Dual use research of concern

Methods

n/a	Involved in the study	
☒	☐	ChIP-seq
☒	☐	Flow cytometry
☒	☐	MRI-based neuroimaging

Eukaryotic cell lines

Policy information about cell lines

Cell line source(s)	Vero cells stably expressing human TMPRSS2 - Markus Hoffmann, Stefan Pöhlmann, German Primate Center – Leibniz Institute for Primate Research, Göttingen, Germany
Authentication	Cells were not authenticated
Mycoplasma contamination	Cells were tested negative for Mycoplasma
Commonly misidentified lines (See ICLAC register)	*Name any commonly misidentified cell lines used in the study and provide a rationale for their use.*

Human research participants

Policy information about studies involving human research participants

Population characteristics	This cross-sectional epidemiological survey targeted all residents of Ischgl/Tyrol irrespective of age and gender. Population of Ischgl, Tyrol, Austria, at time-point of study enrollment (n=1867), including<br>• seasonal immigrant worker with permanent secondary residence (n=250)<br>• children (n=340)
Recruitment	All households in Ischgl were contacted by a postal invitation to attend the study center at a specific time for each household.

Ethics oversight | The ethical committee of the Medical University of Innsbruck approved the study (EC numbers: 1100/2020 and 1111/2020).

Note that full information on the approval of the study protocol must also be provided in the manuscript.
